# Programs to Prepare Siblings for Future Roles to Support Their Brother or Sister with a Neurodevelopmental Disability: a Scoping Review

**DOI:** 10.1007/s40474-023-00272-w

**Published:** 2023-02-21

**Authors:** Linda Nguyen, Jael Bootsma, Marjolijn Ketelaar, Briano Di Rezze, Susan M. Jack, Jan Willem Gorter

**Affiliations:** 1 School of Rehabilitation Science, Faculty of Health Sciences, McMaster University, Hamilton, ON Canada; 2 CanChild Centre for Childhood Disability Research, McMaster University, Hamilton, ON Canada; 3 Centre of Excellence for Rehabilitation Medicine, University Medical Center Utrecht and De Hoogstraat Rehabilitation, Utrecht, the Netherlands; 4 School of Nursing, Faculty of Health Sciences, McMaster University, Hamilton, ON Canada; 5 Department of Health Research Methods, Evidence, and Impact, Faculty of Health Sciences, McMaster University, Hamilton, ON Canada; 6 Offord Centre for Child Studies, Hamilton, ON Canada; 7 Department of Pediatrics, McMaster University, Hamilton, ON Canada

**Keywords:** Children, Youth, Siblings, Disability, Program

## Abstract

**Purpose of Review:**

To identify and map the characteristics and outcomes of programs designed to prepare siblings for their future roles with their sibling with a neurodevelopmental disability.

**Recent Findings:**

Existing programs to support siblings of individuals with a neurodevelopmental disability often focus on providing information about neurodevelopmental disabilities, creating a community for siblings to connect with each other, and connecting siblings to resources and services to support them in their roles. Some programs are offered to the whole family with specific sessions for siblings. While these program descriptions are provided in the literature, there is limited understanding about the impacts and outcomes of these programs on siblings of an individual with a neurodevelopmental disability.

**Summary:**

Fifty-eight articles (published between 1975 and 2020, with > 50% published since 2010) met the inclusion criteria, representing 54 sibling programs from 11 countries. Extracted data represented 1033 (553 females) sibling participants, between 4 and 67 years old. Twenty-seven programs focused on the outcome of knowledge acquisition for the siblings and thirty-one programs focused on the outcome of empowerment for the siblings to teach skills to their sibling with a neurodevelopmental disability. While there is an increasing number of programs for siblings of individuals with a neurodevelopmental disability in the past decade, there is a lack of siblings as co-developers or facilitators. Future research should consider the various roles that siblings can have in programs to address their needs.

**Supplementary Information:**

The online version contains supplementary material available at 10.1007/s40474-023-00272-w.

## Introduction

Worldwide, there are approximately 150 million children and youth under the age of 18 years with a disability including neurodevelopmental disabilities (NDD)[1]. A diagnosis of NDD can include autism spectrum disorder, attention-deficit and hyperactivity disorder, cerebral palsy, Down syndrome, or fetal alcohol spectrum disorder [2, 3]. As children and youth with NDD transition to adulthood, they may experience multiple challenges as they navigate developmental trajectories including exploring options for post-secondary education or accessing health services in the adult care system [4, 5]. Individuals with NDD may seek support from their families as they transition into adulthood, including support with personal care and activities of daily living [4–6]. Many families are well positioned to provide the most optimal support given their history, knowledge, and familiarity of the family member’s care and social needs throughout the individual’s life [6].

In addition to parental support, in families with more than one child, siblings may also emerge as another source of support for an individual with NDD. Every sibling relationship is unique with differing levels of emotional closeness and expectations of each other [7]. Sibling relationships can evolve or change over time based on the needs, roles, and commitments of the whole family [8]. When a sibling has NDD, their sibling may choose to provide support. There are four main types of support: 1) concrete support that includes acts of practical assistance, 2) emotional support that involves acts of empathy, 3) advice support that encompasses acts of provision of information, emotional reassurance, and guidance, and 4) esteem support that includes the reinforcement of the personal worth of an individual [10]. Sometimes, there is an implicit expectation from parents that a sibling will be actively involved in supporting the family member with NDD [9]. Given this, siblings may need support for different roles that they can assume in supporting their brother or sister [10, 11].

Programs are available to support siblings in roles that they may assume. The broad aims of many of these programs include: 1) providing information about NDD, 2) creating sibling communities to connect and share experiences with each other, or 3) connecting siblings to resources and services to assist them in their supporting role [12, 13]. Programs that have been developed have often been tailored to siblings of different ages. Sibshops, for example, was developed in the USA for siblings ages 8 to 13 years old to learn strategies to address situations with their sibling with a disability [12]. Some programs may be targeted for the whole family with specific sessions for siblings to learn about NDD [14] or to learn strategies to connect with the sibling with NDD. In these programs, parents can be trained on how to reinforce these strategies at home [15].

While descriptions of varied programs exist across the literature, there is limited understanding of the impact and outcomes of these programs on siblings of individuals with NDD. This scoping review was conducted to identify and map the characteristics of and outcomes for participants in programs designed to prepare siblings in their future roles to support their brother or sister with NDD. A preliminary search on the JBI Database of Systematic Reviews and Implementation Reports, Cochrane Database of Systematic Reviews, PROSPERO, PubMed, and CINAHL did not identify any reviews on this topic.

### Review Questions

This scoping review was conducted to answering the following two questions:

i. What are the characteristics of programs designed to support siblings of an individual with NDD (e.g., purpose, description, eligibility criteria, length, activities, service provider, and delivery) for siblings of individuals with NDD?
ii. What are the outcomes for siblings of individuals with NDD participating in the programs?

## Methods

This scoping review was conducted according to the Joanna Briggs Institute (JBI) methodology for scoping reviews [16]. The protocol with details of the full search strategy for this review has been published [17]. This report of the scoping review results was written using the Preferred Reporting Items for Systematic Reviews and Meta-Analyses extension for Scoping Reviews (PRISMA-ScR) checklist [18].

### Patient and public involvement

An integrated knowledge translation approach was used in this scoping review, which is an approach to doing research with knowledge users as equal partners with researchers [19]. We partnered with the Sibling Youth Advisory Council (SibYAC) comprised of six young adults who have a sibling with a disability. The SibYAC identified the importance of the research questions addressed in this review and the program outcomes (e.g., knowledge acquisition, skill development, and empowerment). The SibYAC also reviewed the preliminary findings, provided recommendations to interpret the results, and suggested knowledge translation and dissemination activities to share these results with the community.

### Search strategy

An initial limited search was conducted on PsycINFO to identify relevant articles. The text words contained in the titles and abstracts of relevant articles, and the index terms to describe these relevant articles were used to develop a full search strategy for PsycINFO. The full search strategy was then adapted for each included database. The reference list of all included sources of evidence was screened for additional relevant studies. Articles published from database inception to December 20, 2020 were included.

### Information sources

The databases that were searched included PsycINFO, Cumulative Index of Nursing and Allied Health Literature (CINAHL), Sociological Abstracts, Education Resources Information Center (ERIC), EMBASE, Web of Science, MEDLINE (Ovid), and Sport Discus.

### Study of evidence selection

Following the search, all identified citations were collated and uploaded into Covidence, systematic review software (Veritas Health Information, Melbourne, Australia), and duplicates were removed. Following a pilot test, titles and abstracts were screened independently by two reviewers (LN and JB) against the inclusion criteria. Potentially relevant sources were retrieved in full with citation details. The full text of selected studies was assessed in detail against the inclusion criteria by two independent reviewers (LN and JB). Reasons for exclusion of full text studies were recorded. Any disagreements between the reviewers at each stage of the selection process were resolved through discussion or consultation with a third reviewer (MK).

### Inclusion criteria

#### Participants

This review focused on identifying and describing programs with participants who are siblings of an individual with NDD. For this review, NDD is defined as a group of congenital or acquired long-term conditions that resulted from an impairment of the brain and/or neuromuscular system and can lead to functional limitations [2]. In this review, no age limits were applied for the population of the siblings and siblings with NDD and may vary, including children, youth, and adults. In this review, we refer to the siblings of individuals with NDD who participated in the programs as siblings. However, we recognize that these siblings may have had disabilities themselves that were not disclosed in the included studies.

#### Concept

This review included studies that described programs designed to support siblings in their roles. The outcomes of these programs were operationalized to include 1) knowledge acquisition or skill development for the siblings themselves (e.g., knowledge about NDD, sharing and learning experiences about the strengths and challenges in the sibling relationship, development of coping strategies, and problem-solving skills) and 2) empowerment to train siblings to learn skills that they can apply with their sibling with NDD (e.g., how to modify certain behaviors of the sibling with NDD and how to enhance social communication skills in the sibling relationship). Studies about programs that focused only on therapy or support for siblings without reference to support for the individual with NDD have been excluded.

#### Context

The context of this review included all settings that deliver programs for siblings of individuals with NDD, such as school, rehabilitation, healthcare, or community settings, in any country. Only studies published in English were included.

### Types of Sources

This review included all study designs such as experimental and quasi-experimental study designs, randomized controlled trials, non-randomized controlled trials, before and after studies and interrupted time-series studies, single-case studies, descriptive studies, observational studies, qualitative studies, and mixed methods studies.

### Data Extraction

An initial pilot test of the data extraction for approximately 10% of the included studies (*n* = 7) was performed independently by two reviewers (LN and JB) using a data extraction sheet. Based on this initial pilot test, the data extraction tool was modified to provide additional clarity of the information that would be extracted (see Supplementary File 1 for an updated data extraction sheet). For the remaining included studies, data were extracted by one reviewer (LN) and checked by a second reviewer (JB). Disagreements between the reviewers during this check of the extracted data were resolved through discussion or consultation with a third reviewer (MK). The authors of included articles were contacted to request missing or additional data based on the data extraction sheet.

### Data Analysis and Presentation

The extracted data are presented in tabular form that provides a comprehensive overview about sibling programs based on the information outlined in the data extraction form. To address the two research questions of this review, an accompanying descriptive narrative summary is provided in this report.

## Results

**Fig. 1:**
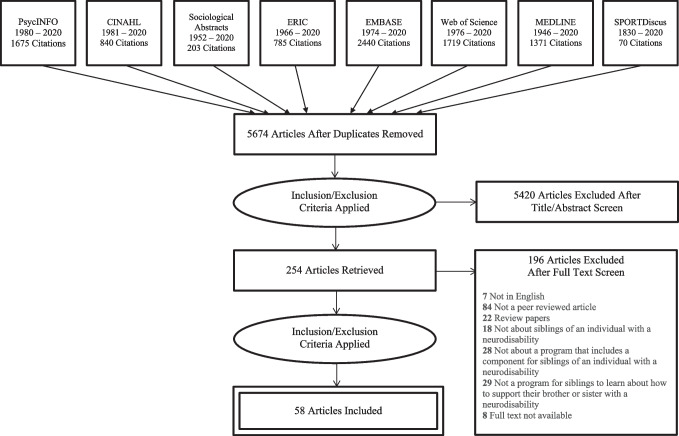
Preferred reporting items for systematic reviews and meta-analyses (PRISMA) diagram outlining the selection process of included studies

There were 5674 non-duplicate articles retrieved through the database searches. No additional studies were identified from the reference lists of included studies. After title and abstract screening, 5420 articles were excluded. There were 254 articles reviewed in full text with a disagreement proportion of 20.8% that were resolved by discussion and/or with a third reviewer, and 196 articles did not meet the inclusion criteria. A total of 58 articles were included in this review (see Fig. 1), representing 54 distinct sibling programs.

### Characteristics of Included Studies

The included articles reported studies from Canada (*n* = 3), USA (*n* = 42), UK (*n* = 4), Ireland (*n* = 1), Turkey (*n* = 1), Norway (*n* = 1), Sweden (*n* = 1), Greece (*n* = 1), Taiwan (*n* = 1), Cambodia (*n* = 1), and Australia (*n* = 2). The articles reported on studies that were conducted from 1975 to 2020, with more than half of the included studies published since 2012 (*n* = 30). Descriptions of included studies are presented in Table 1.

**Table 1:** Description of included studies

Study	Study aim	Type of methodology	Data collection methods	Mean age (SD; standard deviation, range) in years	Diagnosis of participant's siblings
Akers et al. 2018 [20] (USA)	To systematically replicate the results (of script fading by researchers or instructors) with siblings serving as play partners.	Adapted alternating treatment design embedded within a multiple baseline design	Parent report of statements made by the child with NDD.	10 (3.27, 6−14)	Autism spectrum disorder
Brouzos et al. 2017 [21] (Greece)	To evaluate the effectiveness of a psychoeducational group program.	Randomized controlled study	Questionnaires on demographics, knowledge [22], coping and adjustment [23], and strengths and difficulties of the sibling relationship [24].	6−15	Autism spectrum disorder
Burke et al. 2020 [25••] (USA)	To determine the preliminary outcomes of a leadership and support program.	Single-arm, intervention study (a study with an intervention group without a control group)	Questionnaires about participant satisfaction and changes to the program, participation in training activities [26], community or political empowerment [27], advocacy [28], motivation to impact change [29], connectedness to the disability field [30], and open-ended questions of what participants are most hoping to improve or change for individuals with disabilities and their siblings.	36 (21−67)	Intellectual and developmental disability, which includes intellectual disability, autism spectrum disorder, cerebral palsy, Down syndrome
Celiberti et al. 1993 [31] (USA)	To alter the siblings’ behaviour and reinforce their interactions with their sibling with autism spectrum disorder.	Multiple baseline across skills design	Video observation recordings	8.72 (1.09, 8.16−10.25)	Autism spectrum disorder
Chu et al. 2012 [32] (Taiwan)	To examine the effects of peer- and sibling-assisted aquatic program on the interaction behaviors and aquatic skills in children with autism spectrum disorder.	Non-randomized study	Observational video recordings, observations of interaction behaviours using a checklist [33], questionnaire with open-ended and Likert questions, and anecdotal discussions.	7.33 (2.41)	Autism spectrum disorder
Clark et al. 1989 [34] (Canada)	To carry out the following objectives: a) evaluate a group sibling training program designed to enhance social interaction between autistic children and their siblings using a nondidactic problem solving approach; b) to study its effects on various types of interactive behavior; c) to monitor shifts in sibling attitudes; d) to provide social validation within the home setting; and e) to study the temporal generalization of the program.	Multiple-baseline design with follow-up assessment	Observations. Siblings completed questionnaires about home situations, children’s attitudes towards children with an intellectual and/or developmental disabilities [35]. Parents completed questionnaires to assess the extent of positive interactions, conflict, and teaching between siblings at home.		Autism spectrum disorder
Coe et al. 1991 [36] (USA)	To assess and train verbal and nonverbal play responses of children with autism using siblings as primary trainers.	Multiple baseline case study.	Observational recordings written by raters about the performances of the child with NDD and sibling participant.	10 (1, 9−11)	Autism spectrum disorder
Colletti et al. 1977 [37] (USA)	To measure objectively the ability of siblings to effectively work with their brothers or sisters within the home environment. To determine the effectiveness of parents as observers of their own children.	Non-randomized study with an ABAB reversal (withdrawal) design.	Observational recordings.	11 (0.82, 10−12)	Severe neurological impairment and autism spectrum disorder
Craft et al. 1990 [38] (USA)	To explore the effectiveness of siblings as change agents in promoting the functional status of children with cerebral palsy.	Time-series, repeated measures	Measures of change in range of motion and independence in activities of daily living.	9.58 (3.72, 4−17)	Cerebral palsy
Crouthamel 1988 [39] (USA)	To provide time-limited support groups to approximately forty siblings of developmentally delayed children; and to heighten the awareness of community professionals to the special problems and sensitivities of siblings.	Prospective narrative research design	Description of the program presented by the author.	7−13	Developmental disability
D'Arcy et al. 2005 [12] (Ireland)	To evaluate the effectiveness of a sibling program.	Pre-test/post-test design	Questionnaire to assess the sibling’s self-concept [40], as well as a pre-Sibshop interview to evaluate the sibling's knowledge and attitude with regard to their sibling's disability, and to assess their feelings towards their sibling with a disability, their contact with other siblings of children with a disability and their wish to meet them, if they discussed their sibling's disability at home, and their experience of being a sibling within the family.	8−10	Physical or intellectual disability, or a combination of both
Daffner et al. 2020 [41] (USA)	To evaluate the effects of a sibling-mediated intervention on positive and negative social behaviors of young children with attention deficit hyperactivity disorder, and evaluate the implementation integrity and acceptability of this intervention among children with attention deficit hyperactivity disorder.	Non-current multiple baseline design	Video recordings and questionnaires. Parents were asked to complete questionnaires about the behaviour of the sibling with NDD [42], symptoms of ADHD [43], social communications questionnaire [44], and demographic information. Parents were also asked to complete a structured diagnostic interview [45],	10.25 (1.43, 8.33−11.75)	Attention deficit hyperactivity disorder
Doleys et al. 1975 [46] (USA)	To assess the effectiveness of a response cost contingency to modify verbal behaviour.	Non-randomized pre-test post-test case study	Observational recordings in the frequency of repetitions.	19	Intellectual and developmental disorder
Douglas et al. 2018 [47] (USA)	To evaluate a sibling communication program.	Single-subject multiple probe design	Questionnaires. Parents completed questionnaires to gather information about the communication skills of children with complex communication needs [48] and how often the sibling cared for the child with complex communication needs. Questionnaires about demographics and sibling relationships were completed by both parents and siblings [49].	10.44 (3.16, 8.08−14.92)	Speech and motor delay and an emotional disability; Down syndrome; Noonan Syndrome characterized by developmental delays, hypotonia, and vision problems
Dyson 1998 [50] (Canada)	To examine a group support program for children who had a sibling with disabilities and evaluate the effects of the program on siblings.	Program evaluation	Questionnaires of open-ended questions with a forced choice format about what siblings had learned from the program, what program components they enjoyed, and what meeting times they preferred.	7.5−12	Intellectual and developmental disability, autism spectrum disorder, attention deficit disorders sensory impairment, physical disability, learning disabilities and communication disorders, developmental delay, and unspecified disability
Evans et al. 2001 [51] (UK)	To develop and evaluate sibling support groups, designed to help brothers and sisters discuss and explore their relationships with their sibling with learning disabilities.	Qualitative evaluation	Questionnaires. Siblings were asked what they felt they liked and/or disliked about the group, and to make suggestions for the future. Siblings completed tests about family relations [52], self-esteem [53], knowledge about communication and learning disabilities. Questionnaires were also sent to parents.	6−12	Learning disabilities
Ferraioli et al. 2011 [54] (USA)	To systematically replicate an established, adult-mediated intervention with typically developing sibling teachers, and evaluate the program's efficacy in teaching joint attention (JA) skills to children with autism.	Single-subject, multiple probe design	Observation video recordings with an early communication measure [55]. Interviews were conducted with siblings with questions about the quality and quantity of time spent playing with the sibling with NDD.	7.33 (0.96, 6.0−8.33)	Autism spectrum disorder
Fjermestad et al. 2020 [56] (Cambodia)	To describe the level of mental health of sibling and the family, and family communication. To examine the change in mental health symptoms for siblings and parents, and perceived family communication. To examine the usefulness and satisfaction among siblings and parents with the intervention. To evaluate the initial feasibility of the intervention.	Non-randomized pretest post-test design	Translated questionnaires. Both siblings and parents completed questionnaires to measure the sibling mental health status [57]. Each parent completed a questionnaire to assess their mental health [58]. A questionnaire was completed by parents and siblings to measure family communication [59].	12.70 (2.70, 8−21)	Learning problems, intellectual disability, autism spectrum disorder, attention deficit hyperactivity disorder, developmental delay, or Down Syndrome
Fjermestad et al. 2020 [60] (Norway)	To promote sibling well-being.	Randomized controlled trial	Questionnaires and interview. Questionnaires about the mental health of siblings were completed by siblings and parents [57], about family communication were completed by siblings and parents [59], about quality of life by siblings and parents [61], about sibling adaptation by siblings [62], and about sibling knowledge as an interview about the phone by siblings [14].	Not applicable	Neuro-developmental disorder
Gettings et al. 2015 [63] (UK)	To explore the acceptability and feasibility of audio-conference for support groups for siblings, to discuss whether siblings can discuss their concerns through audio-conference, and to explore the impact of support groups.	Non-randomized pretest post-test design.	Semi-structured interview with siblings using the Siblings' Views Questionnaire. Evaluation questionnaire and follow-up interview were completed by siblings. Parents completed questionnaires about the frequency and severity of the symptoms for the sibling with NDD [64]. Siblings and parents completed questionnaires about social, emotional, and behavioural functioning of the siblings [65], and the siblings’ quality of life [66].	8−13	Autism spectrum disorder, ADHD, mood disorder, obsessive compulsive disorder, Down's syndrome, oppositional defiant disorder, visual impairment, multiple anxiety disorders or phobias
Granat et al. 2012 [67] (Sweden)	To study the effectiveness in an outpatient clinical setting of a voluntary group intervention.	Non-randomized pre-test post-test design	Questionnaires were completed by the siblings to assess knowledge about disability [14, 68] and perception of sibling relationship [69].	8−12	Attention deficit hyperactivity disorder, Asperger syndome, autism spectrum disorder, physical disability or intellectual disability.
Hancock et al. 1996 [70] (USA)	To teach older siblings to use two milieu teaching procedures, modeling and mand modeling, with their younger siblings who exhibited language delays. The study examined the effects of the intervention on the older siblings' teaching behavior and on the younger siblings' language use at home during play and snack activities.	Non-randomized study	Observational data, with verbatim transcriptions.	10.67 (1.89, 8−12)	Cerebral palsy, developmental delay of unknown origin, William's syndrome
Hayden et al. 2019 [71] (UK)	To evaluate the Sibs Talk Pilot to help inform Sibs' future work with young siblings	Non-randomized study	Questionnaires. A questionnaire was completed by the teacher to measure behavioural and emotional well-being of the siblings [72, 73], and the children completed a questionnaire about how they feel about school [74, 75].	9.18 years (7−11)	Autism, Down syndrome, hearing impairments or chronic medical condition
James et al. 1986 [76] (USA)	To evaluate a direct prompting training strategy for increasing reciprocal interactions between siblings. To evaluate whether the program would differentially affect initiations of and responses to social interactions. To assess the generalization of interaction skills from dyadic (sibling-sibling) to triadic (sibling-sibling-peer) play groups or across settings.	Multiple-baseline design.	Recordings.	6.83−8.08	Cerebral palsy and intellectual disability
Jones et al. 2020 [77] (USA)	To examine the effects of the support group for siblings of children with autism spectrum disorder.	Randomized controlled trial	Siblings completed questionnaires about mental health and adjustment [78], anxiety [79], support by responding to yes/no questions, and coping [80]. Parents completed questionnaires for each sibling about recent difficulties [81, 82]. Each sibling was evaluated using a rating scale [83].	8.31 years (3.52)	Autism spectrum disorder
Kryzak et al. 2015 [84] (USA)	To evaluate the effects of the program on the siblings’ knowledge about autism spectrum disorder, peer network development, and adjustment as well as interactions between sibling and child with autism spectrum disorder dyads.	Within subject pretest-post-test design.	Questionnaires and observational video recordings. Siblings completed questionnaires about anxiety [79, 85] and knowledge modified from a measure [86]. Behavioural observations of sibling interactions were conducted using video recordings.	4−14 years old	Autism spectrum disorder, which included pervasive developmental disorder not otherwise specified, autism, Asperger’s, or autism spectrum diagnoses
Kryzak et al. 2017 [87] (USA)	To evaluate the effectiveness of combining self-management strategies with an empirically supported social skills curriculum (i.e., Stay-Play-Talk curriculum).	Multiple baseline probe design	Video recordings.	8.5 (2.60, 6−12)	Autism spectrum disorder
Lewandowski et al. 2014 [88] (USA)	To examine the effects of a modification in the procedures for administrating comic strip conversations to address sibling conflict between a child with autism spectrum disorder and his typically developing younger brother.	Case study	Questionnaires. Parents completed questionnaires, including an autism screening measure [89], assessment about social impairments that accompany autism spectrum disorder [90], an assessment of social skills [91], and a measure about the child’s Theory of Mind development [92]. Both the typically developing sibling and the sibling with autism spectrum disorder completed measures about their vocabulary and word retrieval skills [93], verbal mental age [94], and grammatical contrasts such as inflections, function words, and word order [95]. Daily dairies completed by the mother to assess target behaviour. Qualitative interview completed by the parent.	6.17	Autism spectrum disorder or Asperger syndrome
Lobato et al. 1985 [96] (USA)	To assess siblings as the primary therapist in the acquisition of skills particularly important for improved functioning of the disabled child within the family and home (i.e., self-care and domestic skills).	Non-randomized study	Reports from the sibling participant and agreement checker (i.e., mother) about the performance of behaviour.	21	Down's Syndrome
Lobato 1985 [97] (USA)	To develop and evaluate a program model that could address preschool-aged siblings' needs for simple, yet factual explanations, information, and personal-emotional support.	Single-subject experimental design, a multiple-baseline-across-subject groups	Audio recordings.	5.33 (3.75−7)	Hearing loss, left hemiplegia due to a stroke, cerebral palsy, Down syndrome, and intellectual disorder
Lobato et al. 2002 [14] (USA)	To conduct a preliminary evaluation of the intervention on the primary program goals of improving sibling knowledge, sibling adjustment to chronic illness/developmental disability and siblings' sense of connectedness to other children in similar family circumstances.	Non-randomized study	Questionnaires and interview. Interview with sibling to assess knowledge of chronic illness/developmental disability. Siblings and parents completed questionnaires about the adjustment of the sibling to chronic illness/developmental disability [98], sibling connectedness, and global behavioural functioning [99].	9.8 (8−13)	Physical disabilities, autism spectrum disorders, intellectual, medical disorders or combined psychiatric and learning disorders
Lobato et al. 2005 [68] (USA)	To conduct a preliminary evaluation of a program on young siblings' knowledge of their brother or sister's condition, sense of connectedness with others in similar circumstances, and global functioning.	Non-randomized study	Interviews and questionnaires. Interviews with siblings that was structured to assess knowledge of the neurodisability that the sibling had [14]. Siblings completed questionnaires about sibling connectedness, global functioning [99, 100]. Parents completed questionnaires about sibling connected and program satisfaction.	5.7 (4−7)	Autism spectrum disorders including Asperger's disorder, intellectual disability, physical disabilities such as cerebral palsy, medical disorders such as cancer, or dual psychiatric and learning disorders such as Tourette's
McCullough et al. 2011 [101] (USA)	To describe the development of a pilot program to explore the effects of a sibling support group on participants and their families.	Descriptive study	Not applicable. Reports from sibling participants and families were provided.	Not listed.	Autism spectrum disorder and intellectual disability with limited verbal abilities.
McLinden et al. 1991 [102] (USA)	To evaluate the effectiveness of a 6-week support group for siblings of individuals with an intellectual and/or developmental disability.	Randomized controlled trial	Questionnaires and interviews. The sibling completed questionnaires to describe their feelings and behaviors associated with school and home [103], evaluate the effects of works for siblings of children with disabilities [104], and assess the helpfulness of sources of social support available to siblings of children with disabilities [105]. Parents completed a questionnaire to rate the sibling’s behavior [106]. Interviews were conducted with parents.	9.17	Intellectual disability, physical disability, or multiple disabilities
Miller et al. 1976 [107] (USA)	To describe the treatment programs of two families in which siblings were involved as therapeutic agents.	Case report	Video tape recordings.	17.25 (1.92, 15−20) *Note: ages of siblings in the first case study were not provided).	Congenital facial anomaly and slow early development, autism spectrum disorder, intellectual disability
Neff et al. 2017 [108] (USA)	To evaluate the effectiveness of video modeling as an independent teaching tool to teach typically developing siblings how to prompt and reinforce appropriate play during activities with their sibling with autism spectrum disorder. To assess whether increases in independent play occurred with the sibling with autism spectrum disorder.	Non-randomized study	Frequency of occurrence of the target behaviours and scoring whether the occurrence was appropriate or inappropriate.	4.67 (0.94, 4-6)	Autism spectrum disorder
Oppenheim-Leaf et al. 2012 [109] (USA)	To examine whether the teaching interaction procedure would be effective to: teach typically developing siblings to proficiently prompt and reinforce simple social behaviors from their siblings with autism, generalize the skills that typically developing skills learned to other settings without additional prompting, lead to increase in social interactions in the sibling relationship.	Multiple-probe design	Two observers (the teacher and a reliability observer) recorded data with video recordings.	4.67 (0.47, 4−5)	Autism spectrum disorder
Özen 2015 [110] (Turkey)	To examine whether typically developing children will use the social interaction skills in the sibling training package with their siblings with autism spectrum disorder. To examine the effectiveness of sibling-delivered iPad game activities in teaching social interaction skills to siblings with autism spectrum disorder.	Multiple probe design.	Video recordings.	9.67 (0.94, 9−11)	Autism spectrum disorder
Phillips 1999 [111] (USA)	To evaluate the effectiveness of a program.	Randomized controlled trial	Questionnaires. Siblings completed questionnaires about socioemotional adjustment [85], anxiety [79], self-esteem [112], perceived social support [112, 113], stress [114], family functioning [115], and quality of the sibling relationship [49, 69].	11.3 (9−12)	Intellectual disability
Roberts et al. 2015 [116] (Australia)	To evaluate the efficacy of SibworkS in promoting sibling wellbeing.	Randomized controlled trial	Questionnaires. Parents completed questionnaires on behalf of the typically developing siblings about their emotional and behavioural functioning [24], social support that they receive from others [117], quality of the relationship between the typically developing sibling and the sibling with NDD [69], use of avoidant coping responses and approach-based responses [118], global self-esteem [119], behavioural and emotional problems [120], and whether the typically developing siblings attended any other support program. Information about family socioeconomic status was collected based on their postal code [121]. The typically developing siblings listed three things they learned in the final session of the program. Both typically developing siblings and parents completed a questionnaire about their satisfaction with the program that was adapted [122].	9.3 (1.38, 7.5−12.5)	autism spectrum disorder, Angelman's syndrome, Down syndrome, Phelan-McDermid syndrome, global developmental delay, pervasive developmental disorder, intellectual disability, and optic nerve hypoplasia
Roberts et al. 2016 [123] (Australia)	To investigate factors associated with improvement in emotional and behavioural functioning following participation in the program.	Randomized controlled trial	Questionnaires. Parents provided demographic information, including age, gender, diagnosis of the sibling with NDD, and relative age between the typically developing sibling and the sibling with NDD. Parents completed questionnaires about emotional and behavioural functioning of the sibling with NDD [120], and the emotional and behavioural functioning of the typically developing sibling [124]. The family socioeconomic status was collected based on their postal code [121]. Parents indicated whether the typically developing sibling attended any other support services.	9.14 (1.25, 7−13)	Autism spectrum disorder, Crohn's disease, epileptic encephalopathy, attention-deficit hyperactivity disorder, global developmental delay, low muscle tone, dyslexia, dyspraxia, cleft lip, anxiety, depression, oppositional defiance disorder, sensory processing disorder, dysgraphia, Angelman's syndrome, Down’s syndrome, Phelan McDermid syndrome, global developmental delay, pervasive developmental disorder, intellectual disability and optic nerve hypoplasia
Rye et al. 2018 [125] (UK)	To describe the development and qualitative evaluation of a pilot sibling group that aimed to increase siblings' understanding about disabilities, provide a space for peer support and help young people learn skills to help themselves and their siblings at difficult times. To provide a personal account of the experience of typically developing siblings who attended the group sessions.	Qualitative research	Semi-structured interviews with sibling participants.	8−13	Disabilities, including autism spectrum disorder, severe learning disability, chromosomal deletion, cerebral palsy and epilepsy
Schreibman et al. 1983 [126] (USA)	To investigate the effectiveness of a program designed to teach behavior modification procedures to normal siblings of children with autism spectrum disorder.	Multiple-baseline design	Recordings by the researchers by scoring the behavior of the sibling with autism spectrum disorder.	13, 11, 8	Autism spectrum disorder
Sheikh et al. 2019 [15] (USA)	To augment a support group for typically developing siblings of children with autism spectrum disorder with a parent intervention that focused on helping parents learn ways to support interactions between their children, one of whom has autism spectrum disorder.	Non-randomized study	Questionnaires and observations. Parent performance of prompting and reinforcement, and Sibling performance were observed during family play sessions. The typically developing siblings completed a questionnaire to assess the quality of their relationship with their sibling with NDD [84] that was modified from a measure [69], and parents completed a similar measure [84].	5 (0.82, 4−6)	Autism spectrum disorder
Smith et al. 2004 [127] (Canada)	To describe the measures and methods developed, as well results obtained, in a program evaluation of a series of sibling support groups for siblings of children with autism.	Program evaluation	Questionnaires. Parents completed a questionnaire about the internalizing (e.g., depression, anxiety) and externalizing (e.g., hyperactivity) for the typically developing sibling [99]. Typically developing siblings completed measures about how they feel about themselves [40, 103], knowledge about the characteristics and causes of autism, coping and adjustment [23] and psychosocial adjustment specific to the situation of having a sibling with a developmental disorder.	10.63 (2.13, 6.58−16.25)	Autism spectrum disorder or related disorder (e.g., pervasive developmental disorder, Rett disorder, or developmental delay)
Spector et al. 2018 [128] (USA)	To determine whether siblings of children with autism spectrum disorder can learn Natural Language Paradigm and subsequently use it to occasion speech with their brothers. To assess whether children with autism spectrum disorder showed increases in language production following the introduction of sibling-mediated Natural Learning Paradigm. To assess if increases in language production generalized to untrained peers and unfamiliar settings. To measure the presence of behaviors of happiness, joint attention, and play.	Non-concurrent multiple baseline case study design	Video recordings.	9 (1.63, 7−11)	Autism spectrum disorder and speech deficit
Stewart et al. 1987 [129] (USA)	Not applicable.	Descriptive paper	Not applicable.	Not applicable.	Disability.
Stewart et al. 2007 [130] (USA)	To illustrate the process by which a parent-sibling dyad was taught to implement behavioral skills training based social skill training with a sibling with autism spectrum disorder.	Case study design	Video recordings. Parent completed a questionnaire to rate the treatment and outcome [131].	10	Asperger's disorder and attention-deficit/hyperactivity disorder
Swenson-Pierce et al. 1987 [132] (USA)	To evaluate the effectiveness of a sibling training procedure designed to teach school-aged individuals with a disability to perform domestic living skills within the home environment, and to evaluate systematically the success of training procedures by monitoring both the use of instructional techniques by the typically developing sibling and the independent skill performance of the sibling with a disability.	Multiple baseline design	Recordings.	12 (1.41, 10−13)	Intellectual disability, seizures, Down's syndrome, microcephaly, upper extreme spasticity
Trent et al. 2005 [133] (USA)	To evaluate procedures for teaching two responsive interaction strategies (mirroring and verbal responding) to typically developing children in the context of play sessions with their younger siblings with Down syndrome. To investigate the effects of the child-implemented responsive interaction intervention on the communicative behavior of the siblings with Down syndrome.	Multiple-baseline design across subjects and behaviors	Video recordings.	8 (1, 7−9)	Down syndrome
Trent-Stainbrook et al. 2007 [134] (USA)	To replicate a study [133] on teaching older, typically developing siblings how to use responsive interaction strategies (mirroring and verbal responding) in the context of play sessions with their younger siblings with Down syndrome. To investigate the effects of the sibling-implemented responsive interaction intervention on the intentional communicative behavior of the children with Down syndrome. To evaluate the effects of the intervention on both the older siblings and those with Down syndrome in an untrained, generalization setting (i.e., preparing a snack).	Multiple baseline design.	Video recordings.	9.33 (0.47, 9−10)	Down syndrome
Tsao et al. 2006 [135] (USA)	To investigate: a) whether typically developing siblings can learn and use social skills strategies for interacting with their siblings with autism spectrum disorder and b) whether these strategies would result in increased social participation by the siblings with autism spectrum disorder.	Single-subject, multiple-baseline design.	Video recordings.	6.27 (2.83, 4.50−11.17)	Autism and Asperger syndrome
Tsao et al. 2010 [136] (USA)	Not listed. The presentation of a framework for the three-step sibling-mediated social skills intervention.	Descriptive study.	Not applicable.	N/A	Disabilities
Tsao 2020 [137] (USA)	To evaluate the impact of a sibling-mediated social interaction intervention program on social behaviors of children with developmental disabilities.	Single subject, multiple probe design	Video recordings.	5.11 (1.54, 3.33−7.08)	Developmental disabilities, including autism spectrum disorder
Walton et al. 2012 [138] (USA)	To assess whether siblings correctly implemented reciprocal imitation training, whether the training had an effect on engagement with children with autism spectrum disorder, whether the skills were generalizable to other settings, whether observers were able to detect differences in pre- and post-treatment, and to measure the acceptability of the intervention.	Multiple-baseline design	Video recordings.	9.5 (1.71, 8−13)	Autism spectrum disorder
Weinrott 1974 [139] (USA)	To evaluate a training program in behavior modification for siblings of individuals with an intellectual disability. Note that this purpose was not explicitly stated.	Pre-test post-test design	Questionnaires, parental reports, and recordings. Siblings completed questionnaires about behavior situations and knowledge about disability. A play session was recorded between a sibling and a camper.	between 10 and 18 years old.	Intellectual disability
Williams et al. 1997 [140] (USA)	To evaluate the outcomes of a structured, educational, and support group intervention for siblings of children with chronic illness (cancer, cystic fibrosis, diabetes, and spina bifida), including a session with parents about sibling needs; and to describe sibling and parent perceptions of sibling experiences at home.	Pretest-post-test design	Questionnaires. Siblings completed a questionnaire about the knowledge about chronic illness [141]. Parents provided an evaluation rating of the program.	8.5 years (5.57)	Cancer, cystic fibrosis, diabetes, or spina bifida
Williams et al. 2003 [142] (USA)	To assess effects of a full and partial intervention.	Randomized, three-group, repeated measures (panel) design	Questionnaires. Siblings completed measures about their knowledge about illness, social support [143], self-esteem [144], mood [141, 145], attitude toward disability [141, 145]. Parents completed a measure about behavior of the typically developing sibling [146, 147].	11.1 years (2.2)	Cystic fibrosis, diabetes, spina bifida, cancer, and developmental disabilities (which included Down syndrome, autism, traumatic brain injury, or cerebral palsy)

Abbreviation *NDD*, neurodevelopmental disabilities

### Participants

There was a total of 1033 sibling participants (*n* = 399 males and 553 females). Sibling participants ranged in age from 4 to 67 years, with 49 studies with participants younger than 18 years old, three studies with participants 18 years or older, and two studies with a mixture of participants younger and older than 18 years of age (refer to Table 1). There were 22 studies that included participants of siblings with one NDD, including 18 studies that focused on autism spectrum disorder and three studies that focused specifically on attention deficit-hyperactivity order, cerebral palsy, and Down’s syndrome. The remaining studies had participants of siblings with varying health conditions including intellectual and developmental disorders or referred to disabilities as a broad term. The birth order between sibling participants and the sibling with NDD was reported by 37 studies, in which 21 studies had all sibling participants who were older than the sibling with NDD, two studies had sibling participants who were younger than the sibling with NDD, and 12 studies that reported a combination of sibling participants who were older and younger than the sibling with NDD. There were two studies that each reported two twins and one triplet. Detailed participant characteristics in each study are provided in Supplementary File 2.

There were 27 programs that included parental involvement, for example, by completing questionnaires [34, 41, 77, 88, 102, 123, 127, 138] or they were trained to be observers of their child’s performance [37]. Some parents were participants in the program [14, 15, 56, 60, 68, 107, 139, 140, 142], with parent training programs [139], information sessions [142], parent-specific sessions with some parent-sibling sessions [14, 56, 60, 68], or as part of a family training program [15, 107, 130].

### Concept

**Fig. 2:**
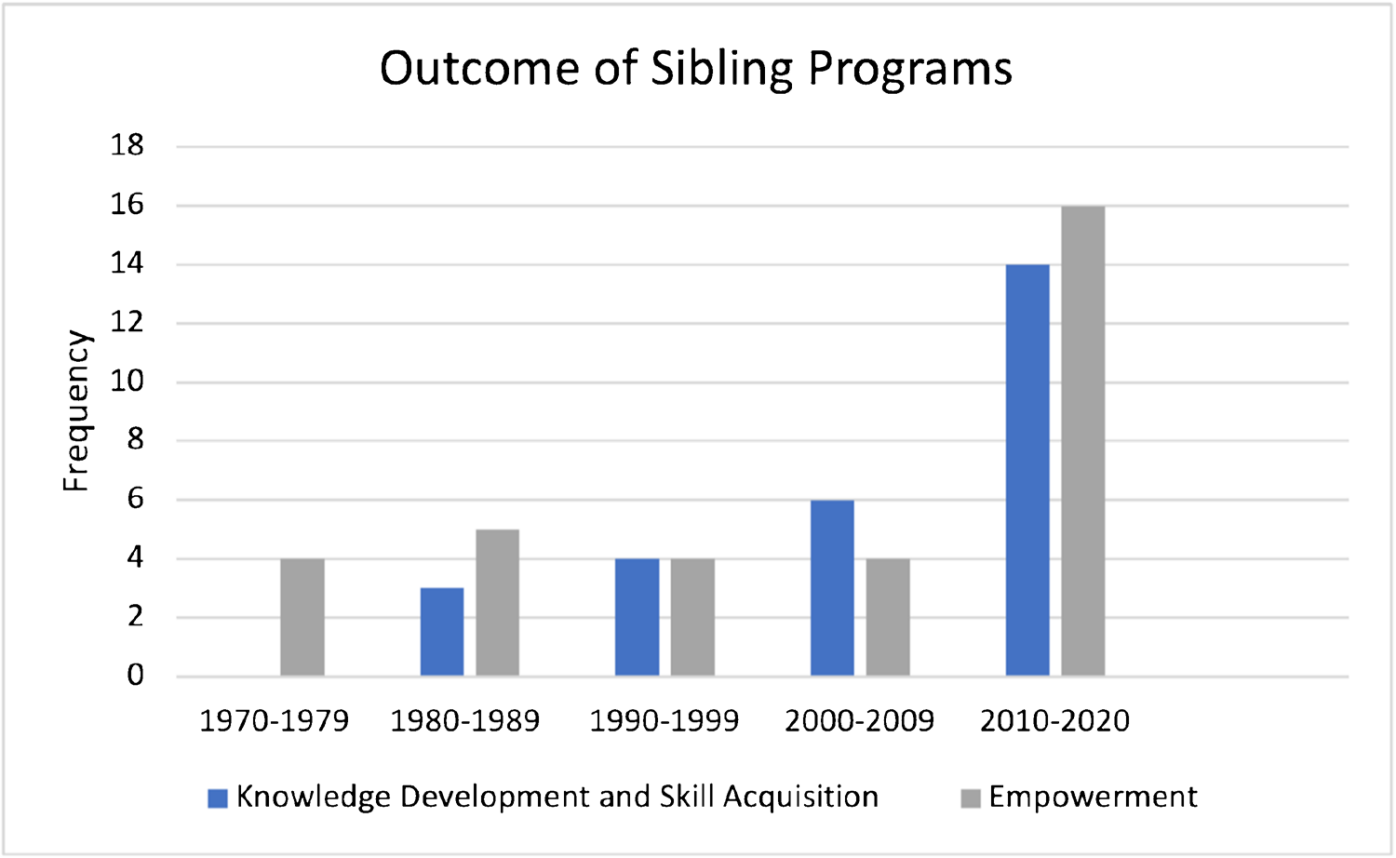
The outcome of sibling programs over time

There were 27 programs that focused on the outcomes of knowledge acquisition or skill development by the siblings for themselves and 31 programs that focused on the outcome of empowering siblings to be trained in specific skills that they can then teach their sibling with NDD. Programs that focused on knowledge acquisition or skill development for siblings were first studied in the 1980s, while programs that focused on empowering siblings have been available since the 1970s (see Fig. 2). For programs about knowledge acquisition or skill development for siblings, the program characteristics are presented in Supplementary File 3 and the outcomes and key findings are presented in Supplementary File 4. For programs about empowering siblings by training them with skills that they can teach to their sibling with NDD, the program characteristics are presented in Supplementary File 5 and the outcomes and key findings are presented in Supplementary File 6.

### Across All Sibling Programs

#### Mode of Delivery

Most programs were delivered in person (*n* = 57). Only one program offered two in-person meetings before incorporating group meetings by telephone [67].

#### Duration and Frequency

The length of the program varied depending on the purpose of the program. For programs that were focused on providing knowledge or skills for the siblings themselves, the sessions were often offered as consecutive weekly sessions, for example, 6 to 10 weeks for approximately half an hour to 2 h [15, 21, 50, 63, 67, 68, 71, 77, 84, 96, 102, 116, 123, 125, 127]. Some programs offered knowledge or skills acquisition for the siblings themselves in a short timeframe, for example, with all sessions in 1 day [56] or 2 days [25••], or for 5 days as part of a summer camp [142]. The programs that trained siblings about specific skills to be applied to the relationship with the sibling with NDD also varied in length. Most programs were delivered with 1–2 sessions per week, between 5 to 22 weeks with sessions ranging from 15 min to 2 h [31, 32, 34, 36, 37, 41, 54, 70, 87, 96, 134].

#### Program Developers

There were 35 studies that described the program developers, with 17 programs focused on providing knowledge or skill development for siblings [12, 14, 15, 21, 25••, 39, 60, 63, 67, 71, 77, 84, 102, 116, 123, 125, 140, 142] and 17 studies that aimed to train siblings to apply skills with their sibling with NDD [32, 41, 46, 47, 54, 70, 87, 88, 96, 126, 128, 130, 134–138]. For programs that aimed to provide knowledge or skill development for the siblings, the developers of the program were primarily from the study authors [14, 21, 60, 84] or organizations that focused to support siblings of individuals with disabilities such as the Sibling Leadership Network in the USA [25••] or Sibs in the UK [71]. There were no studies that explicitly described whether siblings of individuals with NDD were part of the team of developers and the roles that they might have had. Some studies referred to elements of a program based on previous studies about sibling studies [14, 77, 102, 140, 142]. For example, a randomized controlled trial [77] that evaluated a sibling program developed the program based on a study by Kryzak and colleagues [84]. Similarly, programs that aimed to empower and train siblings to teach skills to their sibling with NDD were often based on existing literature or intervention programs [32, 41, 47, 54, 70, 87, 88, 96, 128, 130, 134–138].

#### Program Facilitators

There were 48 studies that described program facilitators [14, 15, 20, 21, 25••, 31, 32, 34, 36, 38, 39, 41, 47, 50, 51, 56, 60, 63, 67, 68, 70, 71, 77, 84, 87, 88, 96, 102, 107–111, 116, 123, 125, 128–130, 132–135, 137–140, 142]. The program facilitators included individuals from a variety of backgrounds including undergraduate students [32, 36], graduate students [14, 15, 21, 31, 68, 77, 84, 116, 123, 130, 138–140], adult siblings of individuals with intellectual and developmental disabilities [25••], healthcare professionals (e.g., social worker, nurse practitioners, nurses, psychologists, and therapists) [34, 39, 51, 56, 63, 67, 87, 102, 107, 116, 123, 128, 140, 142], community center staff [111], special education teachers or staff members at schools [71, 84, 109]. For some programs, the study authors were also the program facilitators [31, 38, 41, 47, 70, 88, 96, 133–135, 138]. Some programs offered opportunities for students to be volunteers and facilitate sessions alongside licensed professionals [84, 111]. While some programs required the facilitators to be licensed professionals, there were programs that also required the facilitators to receive training [56, 71, 84, 134] such as an e-learning course for approximately 1 h with a 2-day workshop [56] or weekly meetings with discussion, coaching, and feedback [134]. One study provided suggestions of facilitators with specific educational backgrounds that might be a good fit to run certain sessions of a program, for example, discussion sessions can be facilitated by teachers, parents, individuals from community organizations, or the siblings themselves [129].

### Context

Among the programs that focused on knowledge acquisition and skill development for siblings, there were 16 programs that described the setting context. These programs were conducted in a variety of settings including at a community center [21, 50, 111], medical center [39, 56, 127, 140] or clinic [97], school [71], or camp [142].

For programs that were focused on training the siblings to learn and apply skills with their siblings with NDD, there were 27 programs that listed the setting context [20, 31, 32, 34, 36–38, 41, 46, 47, 54, 70, 76, 87, 88, 108–110, 126, 128, 132–139]. The majority of these programs was conducted in the participant’s homes [20, 31, 36–38, 41, 47, 54, 70, 76, 87, 88, 108–110, 126, 132–138]. Some programs had sessions that were held in multiple settings. Programs were also held at a community center [32], recreational camp [139], treatment center [108], behavioral management center [128], or at a clinic [34].

### Programs with Outcomes of Knowledge Acquisition and/or Skill Development for the Siblings

#### Purpose

The purpose of the programs was focused on the outcomes of knowledge acquisition and/or skill development for the siblings. To achieve these purposes, there were six programs that provided general information about the developmental or health condition [51, 97, 111, 123, 129, 142]. Other programs provided information tailored to a specific condition, with four programs providing information about autism spectrum disorder [21, 77, 84, 127]. Inherent to many programs to acquire knowledge was a goal of creating opportunities for siblings to connect with peers, for example, to discuss the NDD of their siblings with other siblings [56, 123] or to share their lived experiences of growing up with a sibling with NDD. In addition to knowledge acquisition, several programs included skill development components that included opportunities for siblings to develop coping skills [77, 102] or problem-solving skills to enhance their relationship with their sibling with NDD [21, 67, 77]. For example, programs offered opportunities for siblings to share their lived experiences and learn from each other about how to address certain situations [12, 51, 56, 97, 102].

#### Description of Program Activities

The programs included multiple sessions with a dedicated focus for each session: introductions, structured activities, and concluding session. For the introduction sessions, the content might include icebreaker activities to develop group cohesiveness and rapport [63, 77, 125]. After the introduction sessions, there were multiple sessions with structured activities. These activities included providing knowledge about NDD [50, 56, 60, 63, 67, 68, 84, 116, 123, 125, 140, 142] and learning how to problem-solve and address challenges with a sibling with NDD [14, 15, 51, 63, 77, 84, 125]. One program included activities for adult siblings to learn about disability policy, advocacy, peer support, as well as national, state, and local resources [25••]. Some of the structured activities were focused on further development of group rapport such as recreational and social activities [12, 14, 50, 140] or arts and crafts activities [50, 111, 125]. For the concluding session, some programs ensured that the last session was a celebration, such as with a graduation [14, 68], presentation of diplomas [67], or fun activity chosen by the siblings [125]. Details about the purpose and activities for each program are presented in Supplementary File 3.

#### Program Outcomes and Key Findings

The programs focused on the outcomes of knowledge acquisition and skill development for the siblings. The following information is an overall summary about the key findings from the programs on siblings. The siblings acquired knowledge from these programs in which they experienced an increase in.

understanding about disabilities after the program [12, 14, 21, 51, 63, 67, 71, 84, 97, 125, 127, 140, 142], and some siblings learned about new resources that they could access [25••]. By participating in these programs, siblings identified that they found a support network because they were able to connect with other sibling participants [84, 102, 111, 142]. The siblings also experienced outcomes related to the development of skills for themselves, such as development of self-esteem [51, 111, 142], development of coping skills, [3, 49], decrease in stress [20], improvement in mood [18], and feelings of empowerment [25••]. However, one study identified that the siblings experienced an increase in self-esteem and development of coping strategies but these outcomes were not maintained at follow-up [116]. Details about the program outcomes and key findings for each study are presented in Supplementary File 4.

### Programs to Empower Siblings to Teach Skills to Their Sibling with NDD

#### Purpose

The programs focused on empowerment by training siblings to learn general skills that they can then teach their sibling with NDD. The programs primarily trained siblings to learn skills to interact with their sibling with NDD, for example, how to deal with aggression or improve their communication skills with nonverbal and verbal cues [20, 32, 34, 36, 47, 54, 70, 76, 88, 128, 130, 132–138]. Programs that aimed to enhance social communication skills between the siblings with and without NDD also had specific procedures, such as joint attention intervention [54], milieu teaching procedures [70], natural learning paradigm [128], reciprocal training intervention [138], and script fading procedure [20]. One program focused on addressing sibling conflict, in which both the sibling with and without NDD can learn about social and emotional factors that can help to resolve conflicts [88].

#### Description of Program Activities

The programs included activities to train the siblings to teach their sibling with NDD to learn skills. The following information describes the format and content of these activities to teach these skills to the sibling. The siblings were introduced to the skills using a variety of methods including discussions with the trainer [31, 32, 37, 54, 87, 88, 109, 110, 126, 130, 132–135, 137–139]; teaching materials such as visual text on a PowerPoint, written manual, or handouts [47, 70, 132, 138]; completing homework sheets [34]; reading stories [135–137] or using puppets [136, 137] to illustrate the skill; videos about how the sibling could prompt their sibling with NDD to use the skill and how to praise their sibling for using that skill [41, 47, 108, 110, 126, 128]; modeling the skill with the sibling with NDD by a trainer [31, 76] or parent [107] while the sibling observes; or having a parent explain the skill as a story to the sibling [135]. After the sibling learned the skill, some programs offered opportunities for the sibling to apply how they could teach the skill through verbal practice, questions, and application activities [47, 139], role-play with the trainer with feedback [54, 70, 87, 108, 109, 128, 130, 133, 134, 136, 138], and additional prompts are provided by the trainers during the teaching sessions as needed [54, 133]. Details about the purpose and activities for each study are presented in Supplementary File 5.

While all programs were focused on empowering and training the siblings, there were eight studies that reported about programs with components for other family members. Some programs offered a parent component, such as parent information sheets [116, 123] or an information session [140, 142]. There were four family programs, with sessions for parents and siblings to interact and siblings could share the challenges that they experienced with their parents [14, 56, 60, 68]. There was a program that included both parents and siblings, where parents watched the videotapes that were recorded by the siblings, and there was a discussion about the topics discussed in the videotapes [140]. In addition to a parent component, there was also a component for the siblings with NDD. There were two programs that offered separate sessions that were conducted simultaneously, with a session about social communication for the siblings with autism spectrum disorder and one session for the sibling [77, 84].

#### Program Outcomes and Key Findings

The programs focused on the outcomes of empowerment in which siblings could successfully carry out the skills that they were trained in [31, 32, 34, 37, 41, 46, 54, 76, 87, 96, 108–110, 126, 128, 130, 132, 133, 138]. These skills were primarily focused on the development of positive social behaviors, such as sharing, asking or giving help and compromising [41], setting and monitoring goals [87], or providing tangible reinforcement of a behavior from the sibling with NDD[54, 87]. Other skills that the siblings learned include teaching the sibling with NDD of basic self-care skills [96] or how to communicate with their sibling with NDD [47]. Some siblings stated that the skills were easy to learn [41, 128], while other siblings described that the skills were hard to learn [138]. Some studies indicated that these skills were maintained at follow-up [34, 70, 87, 133, 134]. While learning these skills, the siblings reported increases in self-confidence [38] or feeling enjoyment from spending time with their sibling with NDD [70]. For some siblings, the training was found to be associated with modest positive changes in the interactions between siblings with and without NDD [107, 135]. Two studies found that the skills that siblings learned were generalizable to other settings [87, 108] or with other children with NDD [110]. Details about the program outcomes and key findings are presented in Supplementary File 6.

## Discussion

This review focused on programs to support siblings in their future roles, and these programs aimed to provide knowledge acquisition or skill development for the siblings themselves, or to provide training about specific skills that can be applied with the sibling with NDD. Recent trends about sibling programs were identified in this review with an increase in the availability of sibling programs that focused on knowledge development and skill acquisition as well as empowerment. Starting in 2002, there has been an increase in the involvement of the whole family, including both parents and siblings, in programs. This review identified four studies, referring to two programs, that included the parents and/or caregivers as participants alongside the siblings [14, 56, 60, 68]. In both programs, there were specific sessions for siblings and parents with integrated sibling-parent sessions. Furthermore, there was only one program included in this review that provided a combination of in-person and telephone meetings [67], with all remaining programs that were delivered in-person. In light of the COVID-19 pandemic, some sibling programs have been adapted to be delivered online [148]. Moving forward, there could be considerations about different formats to delivery sibling programs with both online and in-person approaches in order to meet the needs of siblings of individuals with NDD.

In our review of online resources [149], siblings identified in blogs and interviews about the importance of first acquire knowledge about the NDD of their sibling before they could learn specific skills. In addition to knowledge, the siblings also learned about coping skills to address the challenges that they experienced in their sibling relationship. For some siblings, the programs provided a network where siblings could connect and share similar experiences about their relationship with their sibling with NDD. A combination of supports for siblings that come from family, school, peers, and healthcare professionals can be helpful to enhance their ability to cope with certain situations and enhance a positive sibling relationship [150]. It is, therefore, important to consider how programs may need to foster opportunities for siblings to acquire knowledge about NDD and develop skills for themselves, such as coping skills, before providing information about how to take on future supporting roles.

In addition to knowledge and supports for siblings, programs also trained the siblings to learn specific skills to apply with their sibling with NDD. Sibling training programs taught specific skills, such as how to communicate with the sibling with NDD using nonverbal and verbal cues, and were similar to sibling programs described in recent systematic reviews [151•, 152•]. These reviews focused on how siblings have been involved as a playmate, model, or instructor in interventions for children with a disability [151•] or as intervention agents in programs specific to children with autism spectrum disorder [152•]. This review further builds on existing literature by describing the outcome of empowerment when siblings learned about specific skills that they can teach to their sibling with NDD.

### Value of Sibling Programs

Meetings have been held with the SibYAC about the value of sibling programs and the relevance of the review findings to siblings and their families. The findings of this review indicated that siblings and families valued programs to support siblings in acquiring knowledge, developing skills for themselves (e.g., coping strategies), and being trained to apply skills with their sibling with NDD (e.g., using verbal and nonverbal cues). In programs that involved both parents and siblings, [13, 28–30], the activities during these sibling-parent sessions included the siblings creating videos that the parents viewed [14] or for the siblings to share their challenges to their parents and for parents to practice their communication skills [56, 60]. When siblings are preparing for their future roles, there should be planned conversations with the whole family [153]. Despite the important roles that the siblings might have in the future of their sibling with NDD, there are often no formalized plans [154]. Siblings have identified that there should be clear plans in place in order for them to be prepared for their future roles [154–156].

This review identified that sibling programs took place in a variety of settings depending on the purpose of the program. Most programs that were focused on empowering and training siblings about skills to teach their sibling with NDD were conducted at home. Programs that trained the whole family were also conducted at home [15, 107, 130]. It is important to consider how the home environment may be a good fit to conduct certain programs that could involve supporting the sibling and the whole family. The person and the environment can be viewed as a bi-directional transactional process that influences each other [157]. A good fit between the person and environment can positively influence the outcomes of the programs for both the siblings and the siblings with NDD [157]. The SibYAC shared that the environment is not only comprised of the physical home environment but also the family context, similar to what has been identified by other researchers [158]. The siblings have an important role in the family and while they may not physically live in the family home at certain times [159], they should have opportunities and space to discuss roles that they would like to have.

### Strengths and Limitations

A strength of this review is that there was a clear and transparent process to conduct this review, in which protocol was published prior to conducting data analysis. The published protocol and final report were written according to the JBI methodology for scoping reviews [16, 160] and Preferred Reporting Items for Systematic Reviews and Meta-Analyses extension for Scoping Reviews (PRISMA-ScR) checklist [18]. When conducting scoping reviews, it is important that the protocol is developed a priori and that the aims are transparent and reproducible [161]. Another strength of this review is the novel contribution of mapping the availability and type of sibling programs that have been published over time. The growth in the number of available programs for siblings has significance in understanding how siblings can be involved with their sibling with NDD in different ways, such as modifying certain behaviors so that both the sibling and sibling with NDD could communicate with each other.

This review has an additional strength by providing a methodological contribution about how an integrated knowledge translation approach was used to partner with the Sibling Youth Advisory Council throughout the process of conducting this scoping review [19]. This partnership informed the relevance of the research questions, reporting of program outcomes (e.g., knowledge acquisition, skill development, and empowerment), and implications of the findings. The Involvement Matrix [162] was used as a conversation tool about roles and responsibilities, and the SibYAC members preferred to have the roles of being a listener in which they provided with information, a co-thinker in which they were asked to provide an opinion, or an advisor in which they provided (un)solicited advice [162]. Regular updates were provided to the SibYAC at each stage of the review, and the SibYAC shared their perspectives about the implications and value of sibling programs identified in this review.

One limitation of this review is that there were some articles that could not be retrieved in full text, although the corresponding authors and relevant journals were contacted up to three times. However, all non-retrievable articles were published prior to 2013 and may no longer be available. A second limitation is the use of the term NDD in our search strategy, which was defined by healthcare professionals in the context of the traditional medical model [3]. However, since the publication by Morris and colleagues [3], we recognize the shift toward expanding the definition of NDD within a biopsychosocial model that focuses on the development of individuals with NDD within an environment that changes over their life course [163]. There are multiple contextual factors other than the medical diagnosis or impairments that can influence the life of an individual with NDD [164]. A third limitation is that some information was not reported in the articles, such as gender of participants or developers of the program. The synthesis of the findings could only be based on the information that was reported in the studies. A fourth limitation is that the synthesis of extracted data to answer the second question in this review about the outcomes for the siblings of individuals with NDD was only provided descriptively. A fifth limitation is that only programs published in English, and there may be other existing programs for siblings offered in different languages.

### Future Directions

There are several areas for further research that can be conducted to enhance programs for siblings of individuals with NDD. Firstly, it was striking that there was limited information identified in these programs about how siblings can prepare for their future roles. While many programs in this review provided knowledge about NDD to siblings, there was only one program that described providing resources for the siblings [25••]. The siblings participating in programs may wish to access additional resources for their learning. For example, a review of sibling resources was recently synthesized across children’s hospitals, organizations, and treatment centers in Canada that could be shared with siblings [149]. Existing programs could consider how to expand the content of their programs with resources that the siblings may refer to. However, these programs may provide resources to siblings but did not report these resources in their publications. The reporting of these details could be included in future studies.

Secondly, this review highlighted that there were no studies that explicitly described whether siblings of an individual with NDD were co-developers. However, there was one study that identified siblings of an individual with NDD who were facilitators of the program [25••]. Future opportunities could be provided to siblings of individuals with NDD to be engaged as partners in sibling partners with different roles, such as being a co-developer or facilitator, that would be valuable and meaningful [165].

Thirdly, for future sibling programs that are conducted, there could also be an exploration about the evaluations of the effectiveness and social validity of the programs. In a recent systematic review about programs for siblings of children with a disability, the effectiveness of the programs could not be determined due to the variability in the ages of participants, diagnoses of the siblings with a disability, duration of the study, content and structure of the training in the program, and reported outcomes [151•]. A different systematic review of intervention programs that involved siblings [152•] assessed the social validity, including social significance of the goals, social acceptability of the procedures, and social importance of the outcomes. Future programs could measure the social validity using similar methods.

Finally, the majority of programs identified in this review was conducted in the USA. Based on a recent scoping review, there are few programs available for siblings of individuals with NDD in low- and middle-income countries [166]. Although most sibling programs are available in high-income countries, a recent study identified that sibling support provider organizations in Australia, Canada, New Zealand, the UK, and the USA were operated with minimal staffing and funding [148]. While there may be programs offered in other countries besides those included in this review, these programs have not been published. Further research and allocation of funding should be considered about how to offer and publish about these programs to support siblings and the whole family of individuals with NDD across countries.

## Conclusion

This scoping review synthesized the characteristics and outcomes of programs for siblings to support them in their future roles with their sibling with NDD. This review identified that there is an increasing number of sibling programs to provide knowledge or acquisition of skills for the siblings themselves, as well as to train siblings to learn and apply specific skills with their sibling with NDD. Findings from this review can inform future directions for the development and enhancement of sibling programs.

## Supplementary Information

Below is the link to the electronic supplementary material.

Supplementary file1 (DOCX 22.8 KB)

Supplementary file2 (DOCX 92.1 KB)

Supplementary file3 (DOCX 127 KB)

Supplementary file4 (DOCX 112 KB)

Supplementary file5 (DOCX 141 KB)

Supplementary file6 (DOCX 126 KB)

## Data Availability

All data is presented as narrative text and tables to support the findings of this review.
