# Supplementary material for: Programs to Prepare Siblings for Future Roles to Support Their Brother or Sister with a Neurodevelopmental Disability: a Scoping Review
Source: Curr Dev Disord Rep. 2023 Feb 21;10(1):47–79. doi: 10.1007/s40474-023-00272-w (PMC9942034; doi:10.1007/s40474-023-00272-w)
Supplement: Supplementary file 1 — Supplementary file1 (DOCX 22.8 KB) [file 40474_2023_272_MOESM1_ESM.docx]

# Supplementary File 1: Updated data extraction instrument

| **Study Information** | | | |
| --- | --- | --- | --- |
| **Date form completed** | Identify the date that the form was completed (dd/mm/yyyy). | | |
| **Name of person extracting data** | Identify the reviewer who extracted the data for this form. | | |
| **Study title** | Identify the full title and subtitle. | | |
| **Authors** | Identify all authors as follows: “last name, initial of first name.” | | |
| **Author affiliations** | List affiliations of all authors (e.g., program, department, institution). | | |
| **Country** | Identify the country where the study was conducted. | | |
| **Study funding source** | Describe the funding source for the study and role of funders. | | |
| **Possible conflicts of interest** | Identify possible conflicts of interest declared by authors. | | |
| **Population and Setting** | | | |
| **Participant inclusion criteria** | Describe the participant inclusion criteria. | | |
| **Participant exclusion criteria** | Describe the participant exclusion criteria. | | |
| **Participant characteristics** | **Total number** | **Number of males** | **Number of females** |
|  | **Age (years)** | | |
|  | **Education level** | | |
|  | **Race/ethnicity** | | |
|  | **Diagnosis of participant’s siblings** | | |
|  | **Birth order between siblings with and without a neurodevelopmental disability** | | |
|  | **Description of other participants who are not siblings (if applicable)** | | |
| **Methods of participant recruitment** | Identify the different methods of participant recruitment (e.g., social media, word of mouth, participant referral). | | |
| **Setting context** | Describe the environment that the program was implemented (e.g., community, college, university, after school). | | |
| **Methods** | | | |
| **Study aim** | Identify the study aims. | | |
| **Type of methodology** | Describe the methodology that was used. | | |
| **Study steps** | Describe the steps to conduct the study. | | |
| **Data collection methods** | Identify the methods used for data collection (e.g., self-reports, assessment tools, surveys, interviews). | | |
| **Analysis methods** | Identify the methods of analysis (e.g., statistical analyses, qualitative analytic techniques). | | |
| **Program Characteristics** | | | |
| **Name** | Identify the full name and/or abbreviation. | | |
| **Objective(s) for siblings with a neurodevelopmental disability** | State the objectives of the program for siblings with a neurodevelopmental disability. | | |
| **Objective(s) for siblings without a neurodevelopmental disability** | State the objectives of the program for siblings without a neurodevelopmental disability (e.g., training component). | | |
| **Duration and frequency** | Identify the number and length of sessions, as well as the frequency of sessions. | | |
| **Mode of delivery** | Identify the mode of delivery (e.g., online, in-person). | | |
| **Activities** | Identify the activities that were provided in the programs (e.g., weekly meetings, educational workshops, training activities, follow-up activities). | | |
| **Sustainability** | Describe how the program will be sustained (e.g., activities after the study ends). | | |
| **Developers** | Describe the people who developed the program (e.g., number of developers, background, qualifications, skill level). | | |
| **Facilitators** | Describe the people who facilitated the program (e.g., number of facilitators, background, qualifications, education level). | | |
| **Participants’ needs** | Describe the needs (e.g., accessibility and accommodations) of participants in their roles as a sibling while participating in the program. | | |
| **Participants’ goals** | Describe the self-reported goals of participants in the program and the progress towards achieving these goals. | | |
| **Study Results** | | | |
| **Outcomes** | Report on the outcomes that were measured in the study. | | |
| **Key findings for the siblings with a neurodevelopmental disability** | Describe the key findings for the siblings with a neurodevelopmental disability (e.g., a summary in the discussion section). | | |
| **Key findings for the siblings without a neurodevelopmental disability** | Describe the key findings for the siblings without a neurodevelopmental disability (e.g., a summary in the discussion section). | | |
| **Study reference** | Provide the full reference for the study. | | |
| **Other studies of interest** | Identify other studies of interest based on the reference list. | | |
