# Supplementary material for: Programs to Prepare Siblings for Future Roles to Support Their Brother or Sister with a Neurodevelopmental Disability: a Scoping Review
Source: Curr Dev Disord Rep. 2023 Feb 21;10(1):47–79. doi: 10.1007/s40474-023-00272-w (PMC9942034; doi:10.1007/s40474-023-00272-w)
Supplement: Supplementary file 2 — Supplementary file2 (DOCX 92.1 KB) [file 40474_2023_272_MOESM2_ESM.docx]

**Supplementary File 2.** Description of participant characteristics.

| **Study** | **Participant inclusion criteria** | **Total number** | **Number of males** | **Number of females** | **Mean age (SD; standard deviation, range) in years** | **Diagnosis of participant's siblings** | **Birth order between the sibling compared to the sibling with NDD** | **Description of other participants who are not siblings (if applicable)** |
| --- | --- | --- | --- | --- | --- | --- | --- | --- |
| Akers et al. 2018 (1) | Siblings of child who: a) previously or currently attending a university-based behavioral preschool (aka diagnosis of ASD); b) ability to emit at least 3 word phrases; c) had a generalized imitation repertoire; d) engaged in low levels of destructive behavior; and e) played with toys appropriately but rarely commented during play. | 3 | 1 | 2 | 10 (3.27, 6-14) | Autism spectrum disorder | All siblings were older than the sibling with autism spectrum disorder | Not applicable |
| Brouzos et al. 2017 (2) | Siblings of a child with autism spectrum disorder. | 38 | Experi-  mental group: 10 boys  Control group: 8 boys | Experimen-tal group: 12 girls  Control group: 8 girls | 6-15 | Autism spectrum disorder. | Not listed | Not applicable |
| Burke et al. 2020 (3) | Not mentioned. Siblings of individuals with intellectual and developmental disabilities. | 21 | 6 | 15 | 36 (21-67) | Intellectual and developmental disability, which includes intellectual disability, autism spectrum disorder, cerebral palsy, Down syndrome | Not listed | Not applicable |
| Celiberti et al. 1993 (4) | Siblings who are at least five years old and not more than one year younger or six years older than the child with autism spectrum disorder. The child with autism spectrum disorder had to engage in little, if any, cooperative play. Siblings should have no known behavioral or academic problems. | 3 | 0 | 3 | 8.72 (1.09, 8.16-10.25) | Autism spectrum disorder | Siblings were older | Not applicable |
| Chu et al. 2012 (5) | Participants were recruited from similar neighbourhoods to the participants with autism spectrum disorder. They were eligible to participate in the study if they had no history or ongoing medical treatment and met the inclusion criteria for each group.  For the sibling-assisted group, both the child with autism spectrum disorder and their sibling were recruited if they (a) were healthy, (b) were between ages 7 and 12 years old, (c) lived together, and (d) were able to follow instructions and the requirements of the sibling-assisted aquatic program. For the peer-assisted group and control group of children, they were recruited if they were (a) healthy, (b) within 7-12 months of age as the other participants had to be, and (c) able to follow the requirements of either the peer-assisted aquatic program or control instructions. | 7 | 2 | 5 | 7.33 (2.41) | Autism spectrum disorder | Not listed | Participants in the peer-assisted and control group |
| Clark et al. 1989 (6) | Not mentioned. Siblings of a child with autism spectrum disorder, which was assessed as meeting Diagnostic and Statistical Manual of Mental Disorders, Third Edition (7), criteria for autism spectrum disorder by two independent diagnosticians. | 3 | 1 | 2 |  | Autism spectrum disorder | Pair 1: Sibling younger  Pair 2: Sibling older  Pair 3: Sibling older | Parents completed questionnaires |
| Coe et al. 1991 (8) | Not listed. | 2 | 2 | 0 | 10 (1, 9-11) | Autism spectrum disorder | Siblings were older | Not applicable |
| Colletti et al. 1977 (9) | Not listed. | 3 | 2 | 1 | 11 (0.82, 10-12) | Severe neurological impairment and autism spectrum disorder | Siblings were older | Parents were trained to be observers of their children's performance |
| Craft et al. 1990 (10) | Siblings of children with cerebral palsy, ages 4-17 years old, were available for pre-testing, intervention and post-testing, and had given their consent. | 31 | 15 | 16 | 9.58 (3.72, 4-17) | Cerebral palsy | A mixture of older and younger siblings | Not applicable |
| Crouthamel 1988 (11) | Not listed. | 12 | 9 | 3 | 7-13 | Developmental disability | Not listed | Networking community professionals were involved in a separate part of the project |
| D'Arcy et al. 2005 (12) | Siblings between 8 and 10 years with a sibling with a disability. | 16 | 11 | 5 | 8-10 | Physical or intellectual disability, or a combination of both | 50% older siblings, 50% younger siblings | Not applicable |
| Daffner et al. 2020 (13) | Not listed. However, the participant inclusion criteria was provided for the child with attention deficit hyperactivity disorder. The sibling had to be at least 7 years old, with an age gap between the siblings that could not exceed 6 years. | 3 | 1 | 2 | 10.25 (1.43, 8.33-11.75) | Attention deficit hyperactivity disorder | Siblings were older | Parent completed questionnaires. |
| Doleys et al. 1975 (14) | Not listed. | 1 | 0 | 1 | 19 | Intellectual and developmental disorder | Siblings were older | Not applicable. |
| Douglas et al. 2018 (15) | Siblings with typical development, between 7 to 15 years old, has a sibling with a communication disability who requires the use of alternative communication such as sign, gestures, computer systems, or picture symbols. When there was more than one sibling who met the eligibility requirements, the parent was instructed to select the sibling who played most frequently with the child with complex communication needs. | 3 | 1 | 2 | 10.44 (3.16, 8.08-14.92) | Speech and motor delay and an emotional disability; Down syndrome; Noonan Syndrome characterized by developmental delays, hypotonia, and vision problems | Siblings were older | Not applicable. |
| Dyson 1998 (16) | Siblings of a sibling with a disability. | 40 | 24 | 16 | 7.5-12 | Intellectual and developmental disability, autism spectrum disorder, attention deficit disorders sensory impairment, physical disability, learning disabilities and communication disorders, developmental delay, and unspecified disability | Not listed. | Not applicable. |
| Evans et al. 2001 (17) | Not listed. | 28 | 9 | 19 | 6-12 | Learning disabilities | Not listed. | Not applicable. |
| Ferraioli et al. 2011 (18) | Siblings of a sibling with a diagnosis of autism spectrum disorder according to Diagnostic and Statistical Manual of Mental Disorders, Fourth Edition (19), criteria, as determined by an unaffiliated professional. | 4 | 4 | 0 | 7.33 (0.96, 6.0 – 8.33) | Autism spectrum disorder | Siblings were older | Not applicable |
| Fjermestad et al. 2020 (20) | Sibling of an individual with a neurodevelopmental disorder. | 52 | 66% | 44% | 12.70 (2.70, 8-21) | Learning problems, intellectual disability, autism spectrum disorder, attention deficit hyperactivity disorder, developmental delay, or Down Syndrome | Not listed | Parents as participants in the program |
| Fjermestad et al. 2020 (21) | 1) Being the sibling of a child diagnosed with a neurodevelopmental disorder who is aged 0 to 18 years and who receives specialist and/or municipal health services; 2) ages 8-16 years; and 3) one parent able to attend the intervention. | Anticipated enrollment of 288 participants. | Not applicable | Not applicable | Not applicable | Neuro-developmental disorder | Not applicable. | Parents as participants in the program |
| Gettings et al. 2015 (22) | Not listed.  Siblings of patients being treated at the Centre for Interventional Pediatric Psychopharmacology, a national specialist Child and Adolescent Mental Health Service in the UK. All patients had complex neurodevelopmental disorders involving at least two co-morbid conditions such as autism spectrum disorder, attention deficit hyperactivity disorder, obsessive compulsive disorder, oppositional defiant disorder or anxiety disorders. | 6 | 1 | 5 | 8-13 | Autism spectrum disorder, ADHD, mood disorder, obsessive compulsive disorder, Down's syndrome, oppositional defiant disorder, visual impairment, multiple anxiety disorders or phobias | Siblings without NDD were younger than siblings with a neuro-disability. | All siblings were accompanied by parents. Five mothers and one father took part. |
| Granat et al. 2012 (23) | Siblings in families of children registered at an outpatient habilitation centre in mid-Sweden, ages 8-12 years old with no diagnosed disability. | 54 | 21 | 33 | 8-12 y | Attention deficit hyperactivity disorder, Asperger syndome, autism spectrum disorder, physical disability or intellectual disability. | 31 siblings were older | Not applicable. |
| Hancock et al. 1996 (24) | The sibling was (a) at least 8 years of age, (b) chronologically older than the sibling with NDD, and (c) willing to participate in the study. The sibling with NDD was (a) between the ages of 4 and 8 years, (b) functioning in the mild to moderate range of an intellectual and developmental disorder, (c) spontaneously using one- to three-word utterances, and (d) socially responsive to initiations made by his sibling. | 3 | 0 | 3 | 10.67 (1.89, 8-12) | Cerebral palsy, developmental delay of unknown origin, William's syndrome | Siblings were older | Not applicable |
| Hayden et al. 2019 (25) | Not listed. Sibling of an individual with a disability. | 55 | Number not listed, 45.5% | Number not listed. 54.5% | 9.18 years (7-11) | Autism, Down syndrome, hearing impairments or chronic medical condition | Not listed | Not applicable |
| James et al. 1986 (26) | Not listed. Sibling of an individual with a disability. | 3 | 1 | 2 | 6.83-8.08 | Cerebral palsy and intellectual disability | Siblings were older  . | TD peers nonhandicapped peers friends of the siblings participated, and parent ratings were collected |
| Jones et al. 2020 (27) | Sibling of an individual with autism spectrum disorder. | 54 (24 in the control group, 30 in the support group) | 24 | 30 | 8.31 years (3.52) | Autism spectrum disorder | Control group: 15 siblings were older, 13 were younger, and 2 were of the same age.    Support group: 12 siblings were older than their siblings with ASD, 9 were younger, and 3 were of the same age. | Parents completed measures |
| Kryzak et al. 2015 (28) | Sibling (i.e., with no known diagnosis of autism spectrum disorder) of a child who has a diagnosis of autism spectrum disorder from an outside source (per DSM-IV-TR; American Psychiatric Association, 2000). | 15 | 9 | 6 | 4-14 years old | ASD, which included pervasive developmental disorder not otherwise specified, autism, Asperger’s, or autism spectrum diagnoses | Not listed. | Sibling with ASD. |
| Kryzak et al. 2017 (29) | Average performance on the Stanford-Binet Intelligence Scales - Fifth Edition; and nonclinical scores on the parent-completed Child Behavior Checklist.    Sibling of a sibling with autism spectrum disorder, parent report of a diagnosis of autism spectrum disorder made by practitioners not associated with the study. | 4 | 1 | 3 | 8.5 (2.60, 6-12) | Autism spectrum disorder | Not listed | Four sibling dyads participated to provide a comparison with interactions between the sibling and sibling with ASD dyads for social validity purposes. |
| Lewandowski et al. 2014 (30) | Not listed. Sibling of a child with autism spectrum disorder. | 1 | 1 | 0 | 6.17 | Autism spectrum disorder or Asperger syndrome | Sibling was younger | The children's birth mother completed measures |
| Lobato et al. 1985 (31) | Not listed. Sibling of a sibling with NDD. | 1 | 0 | 1 | 21 | Down's Syndrome | Sibling was older | Discussions were held with the child's parents, two sisters, and maternal grandmother. |
| Lobato 1985 (32) | Not listed. Sibling of a sibling with a disability. | 6 | 3 | 3 | 5.33 (3.75-7) | Hearing loss, left hemiplegia due to a stroke, cerebral palsy, Down syndrome, and intellectual disorder | 5 siblings were older | Parent recordings of sibling interactions at home |
| Lobato et al. 2002 (33) | Not listed. Siblings, ages 8 to 13 years old, of a sibling with a chronic illness/developmental disability. | 54 | 24 | 30 | 9.8 (8-13) | Physical disabilities, autism spectrum disorders, intellectual, medical disorders or combined psychiatric and learning disorders | 57% of siblings were older | Parents participated in the program and completed measures |
| Lobato et al. 2005 (34) | Not listed. Siblings (ages 4-7 years) of a sibling with a chronic illness or developmental disability | 43 | 17 | 26 | 5.7 (4-7) | Autism spectrum disorders including Asperger's disorder, intellectual disability, physical disabilities such as cerebral palsy, medical disorders such as cancer, or dual psychiatric and learning disorders such as Tourette's | 51% of the siblings were older | Parents participated in the program and completed measures |
| McCullough et al. 2011 (35) | Not listed. | 3 | 1 | 2 | Not listed. | Autism spectrum disorder and intellectual disability with limited verbal abilities. | Not listed | Not applicable |
| McLinden et al. 1991 (36) | Sibling of a sibling with a disability. | 6 | 1 | 5 | 9.17 | Intellectual disability, physical disability, or multiple disabilities | Not listed. | Parents completed a questionnaire, and mothers were interviewed |
| Miller et al. 1976 (37) | Not listed. Sibling of a sibling with NDD. | 8 | 5 | 3 | 17.25 (1.92, 15-20)  *Note: ages of siblings in the first case study were not provided). | Congenital facial anomaly and slow early development, autism spectrum disorder, intellectual disability | Siblings were older | Parents were participants in the program |
| Neff et al. 2017 (38) | Not listed. Sibling of a sibling with autism spectrum disorder. | 3 | 1 | 2 | 4.67 (0.94, 4-6) | Autism spectrum disorder | Twins, one sibling was older, and one sibling was younger | Not applicable |
| Oppenheim-Leaf et al. 2012 (39) | Not listed. Sibling of a sibling with autism spectrum disorder. | 3 | 2 | 1 | 4.67 (0.47, 4-5) | Autism spectrum disorder | Two siblings were older and one sibling was younger | Not applicable |
| Özen 2015 (40) | Pre-requisite skills that siblings should have are: (i) volunteer for participation in the study, (ii) be a student in primary school, (iii) be independent in using an iPad, (iv) be able to play the selected iPad game after one or two rehearsals, (v) be able to understand the visual material and verbal presentation regarding the sibling training session, (vi) be able to interact socially with peers and adults, and (vii) be available to participate in the study at least twice a week. Playing with the iPad should be one of the favorite leisure activities of the siblings as well.    The following pre-requisite skills were for the siblings with autism spectrum disorder: (i) have a diagnosis of autism spectrum disorder, (ii) be able to follow directions, (iii) being able to pay attention to visual or verbal stimulus for at least 5 minutes, (iv) be able to keep eye contact, (v) be able to watch a scene on the computer or iPad for at least 2 minutes, (vi) not have any experience of systematical training with the iPad, and (vii) be able to slide their index finger on the iPad screen and press with their finger. | 3 | Not listed. | Not listed. | 9.67 (0.94, 9-11) | Autism spectrum disorder | All siblings were older | Mothers were asked to complete a questionnaire |
| Phillips 1999 (41) | Siblings of children with identified developmental disabilities. | 180 | 72 | 108 | 11.3 (9-12) | Intellectual disability | Not listed | Not applicable |
| Roberts et al. 2015 (42) | Sibling had to be aged between 8 and 12 years with 6 months allowance on either side; both sibling and parent consent had to be obtained; and siblings themselves could not have a significant disability or chronic health condition. | 42 (22 in intervention group, 20 in waitlist control group) | 25 | 17 | 9.3 (1.38, 7.5-12.5) | autism spectrum disorder, Angelman's syndrome, Down syndrome, Phelan-McDermid syndrome, global developmental delay, pervasive developmental disorder, intellectual disability, and optic nerve hypoplasia | 21 siblings were older | Parents completed outcome measures on behalf of participants. The parent component of SibworkS consisted of Parent Information Sheets provided in the manual. These summarized the the main theme of each session and the rationale for exploring it. They also offer ‘Parent Tips’, which are a mixture of practical tips and advice for encouraging further discussion about the session theme. |
| Roberts et al. 2016 (43) | Siblings aged between 8 and 12 with 6 months allowance either side, could not have a disability, and have a sibling with a disability (i.e., special requirements for their care as a result of a physical or developmental challenge). | 36 | 14 | 22 | 9.14 (1.25, 7-13) | Autism spectrum disorder, Crohn's disease, epileptic encephalopathy, attention-deficit hyperactivity disorder, global developmental delay, low muscle tone, dyslexia, dyspraxia, cleft lip, anxiety, depression, oppositional defiance disorder, sensory processing disorder, dysgraphia, Angelman's syndrome, Down’s syndrome, Phelan McDermid syndrome, global developmental delay, pervasive developmental disorder, intellectual disability and optic nerve hypoplasia | Not listed. | Parent component, in which parent information sheets were provided in the manual. These summarize the main theme of each session (1–6 above) and the rationale for its incorporation into the program. They also offer ‘Parent Tips’, which are a combination of practical tips as well as advice for encouraging further discussion about the session themes. |
| Rye et al. 2018 (44) | Siblings of an individual with a disability known to the Ealing Service for Children with Additional Needs. | 4 | 0 | 4 | 8-13 | Disabilities, including autism spectrum disorder, severe learning disability, chromosomal deletion, cerebral palsy and epilepsy | Not listed. | Not applicable. |
| Schreibman et al. 1983 (45) | Siblings of a sibling with autism spectrum disorder that was diagnosed by at least two outside agencies not associated with the research projects. | 3 | 1 | 2 | 13, 11, 8 | Autism spectrum disorder | Siblings were older | Not applicable |
| Sheikh et al. 2019 (46) | Siblings had to have a sibling with autism spectrum disorder diagnosed by a professional not associated with this research, with a parent interested in parent-sibling training. | 3 | 1 | 2 | 5 (0.82, 4-6) | Autism spectrum disorder | One sibling-dyad were twins, and two sibling-dyads where the siblings were younger | Parents were involved in the parent-sibling training that involved the whole family |
| Smith et al. 2004 (47) | Not listed. Siblings of families who attended the TRE-ADD (Treatment, Research, and Education for Autism and Developmental Disorders) program at Thistletown Regional Centre, of a sibling with autism or related disorder (e.g., pervasive developmental disorder, Rett disorder, or developmental delay). | 26 | 12 | ~~14~~ | 10.63 (2.13, 6.58-16.25) | Autism spectrum disorder or related disorder (e.g., pervasive developmental disorder, Rett disorder, or developmental delay) | 14 siblings were older, 12 siblings were younger | Parents completed questionnaires |
| Spector et al. 2018 (48) | Siblings had to have a sibling with autism spectrum disorder diagnosed by two licensed psychologists from two independent agencies not affiliated with this study according to the Diagnostic and Statistical Manual of Mental Disorders, Fifth Edition (49) and also displayed a speech deficit. | 3 | 1 | 2 | 9 (1.63, 7-11) | Autism spectrum disorder and speech deficit | Siblings were older | Not applicable |
| Stewart et al. 1987 (50) | Not a study with participants. However, the program is for siblings of individuals with a disability. | Not applicable. | Not applicable. | Not applicable. | Not applicable. | Disability. | Not applicable. | Not applicable. |
| Stewart et al. 2007 (51) | Not listed. Sibling of a sibling with autism spectrum disorder (specifically Asperger's disorder) | 1 | 0 | 1 | 10 | Asperger's disorder and attention-deficit/hyperactivity disorder | Sibling was the same age (both part of triplets) | The sibling participant assisted mother during training. |
| Swenson-Pierce et al. 1987 (52) | Older siblings of a sibling with a disability and a need for the sibling with a disability to acquire domestic skills. | 3 | 1 | 2 | 12 (1.41, 10-13) | Intellectual disability, seizures, Down's syndrome, microcephaly, upper extreme spasticity | Siblings were older | Not applicable |
| Trent et al. 2005 (53) | Siblings between 6-12 years old, were chronologically older than the siblings with Down syndrome, and the siblings with Down syndrome were between 5-11 years old with significant language delays. | 2 | 0 | 2 | 8 (1, 7-9) | Down syndrome | Siblings were older | Not applicable |
| Trent-Stainbrook et al. 2007 (54) | Siblings between 6-12 years old, have a younger sibling with Down syndrome between 5-11 years old. | 3 | 1 | 2 | 9.33 (0.47, 9-10) | Down syndrome | Siblings were older | Not applicable |
| Tsao et al. 2006 (55) | Not listed. Siblings were between 4 to 11 years of age of a sibling between 3 and 6 years of age diagnosed with autism or another autistic spectrum disorder (i.e., pervasive developmental disorder, not otherwise specified) by a physician, psychologist, or diagnostic clinic staff member using the criteria from the Diagnostic and Statistical Manual of Mental Disorders-Fourth Edition (19) | 4 | 2 | 2 | 6.27 (2.83, 4.50-11.17) | Autism and Asperger syndrome | Two siblings were younger, and two siblings were older | Parents would stay with their children throughout the experiment. During the baseline, parents were asked to act as they usually did with their children. In the intervention phases, the parents watched how the researcher taught a lesson to the sibling and could offer suggestions that might help the sibling understand the lesson. Parents could also offer verbal prompts to both of siblings in the dyad during the intervention and maintenance phases. At the end of each play session in intervention, parents were asked to offer feedback on the lesson plan. |
| Tsao et al. 2010 (56) | N/A | N/A | N/A | N/A | N/A | Disabilities | N/A | N/A |
| Tsao 2020 (57) | Not listed. TD brothers of a brother with a developmental disability evaluated by a certified psychologist or clinician. | 3 | 3 | 0 | 5.11 (1.54, 3.33-7.08) | Developmental disabilities, including autism spectrum disorder | Two siblings were older and one sibling was younger | Parents were present at each session but did not engage in the interactions |
| Walton et al. 2012 (58) | Not listed. Sibling of a sibling with ASD diagnosed by a professional with expertise in autism and exceeded the cutoff scores for “autism spectrum” on the social, communication, and social + communication algorithms of the Autism Diagnostic Observation Schedule-Module 1 (59). | 6 | 2 | 4 | 9.5 (1.71, 8-13) | Autism spectrum disorder | Siblings were older | Parents reported on the satisfaction with the program |
| Weinrott 1974 (60) | Siblings ages 10-18 of a sibling with an intellectual disability who were living in the Boston area, since pre-camp training would be held at Harvard University and The Walter E. Fernald State School. | 18 | Not listed. | Not listed. | between 10 and 18 years old. | Intellectual disability | Not listed. | Parents participated in the training program. |
| Williams et al. 1997 (61) | Not listed. Siblings of children with diagnoses of cancer, cystic fibrosis, diabetes, or spina bifida. | 22 | 9 | 13 | 8.5 years (5.57) | Cancer, cystic fibrosis, diabetes, or spina bifida | Not listed. | Parents attended a discussion about issues related to sibling adjustment. |
| Williams et al. 2003 (62) | Sibling who was closest in age to the sibling with NDD. | 79 | 41 | 38 | 11.1 years (2.2) | Cystic fibrosis, diabetes, spina bifida, cancer, and developmental disabilities (which included Down syndrome, autism, traumatic brain injury, or cerebral palsy) | Not listed. | Parents participated in sessions and completed questionnaires. |

Abbreviations: N/A, not applicable; NDD, neurodevelopmental disabilities.

*Note 1: For the purpose of this review, the gender of participants was reported instead of sex. The reporting of gender aligns with its definition as a socially constructed role (63), and siblings may choose to have different roles while participating in programs.

**Note 2: The mean age and standard deviation was manually calculated, if the authors of included studies provided all available data for each individual participant.

**References**

1. Akers JS, Higbee TS, Pollard JS, Reinert KS. Sibling-implemented script fading to promote play-based statements of children with autism. Behav Anal Pract. 2018;11(4):395–9.

2. Brouzos A, Vassilopoulos SP, Tassi C. A psychoeducational group intervention for siblings of children with autism spectrum disorder. J Spec Gr Work. 2017;42(4):274–98.

3. Burke MM, Lee CE, Carlson SR, Arnold CK. Exploring the preliminary outcomes of a sibling leadership program for adult siblings of individuals with intellectual and developmental disabilities. Int J Dev Disabil. 2018;0(0):1–8.

4. Celiberti DA, Harris SL. Behavioral intervention for siblings of children with autism: A focus on skills to enhance play. Behav Ther. 1993;24(4):573–99.

5. Chu CH, Pan CY. The effect of peer- and sibling-assisted aquatic program on interaction behaviors and aquatic skills of children with autism spectrum disorders and their peers/siblings. Res Autism Spectr Disord. 2012;6(3):1211–23.

6. Clark ML, Cunningham LJ, Cunningham CE. Improving the social behavior of siblings of autistic children using a group problem solving approach. Child Fam Behav Ther. 1989;11(1):19–33.

7. American Psychiatric Association. Diagnostic and statistical manual of mental disorders. 3rd ed. 1980.

8. Coe DA, Matson JL, Craigie CJ, Gossen MA. Play skills of autistic children: Assessment and instruction. Child Fam Behav Ther. 1991;13(3):13–40.

9. Colletti G, Harris SL. Behavior modification in the home: Siblings as behavior modifiers, parents as observers. J Abnorm Child Psychol. 1977;5(1):21–30.

10. Craft MJ, Lakin JA, Oppliger RA, Clancy GM, Vanderlinden DW. Siblings as change agents for promoting the functional status of children with cerebral palsy. Dev Med Child Neurol. 1990;32(12):1049–57.

11. Crouthamel CS. Siblings of handicapped children: A group support program. Early Child Dev Care. 1988;37:119–31.

12. D’Arcy F, Flynn J, McCarthy Y, O’Connor C, Tierney E. Sibshops. An evaluation of an interagency model. J Intellect Disabil. 2005;9(1):43–57.

13. Daffner MS, DuPaul GJ, Kern L, Cole CL, Cleminshaw CL. Enhancing social skills of young children with ADHD: Effects of a sibling-mediated intervention. Behav Modif. 2020;44(5):698–726.

14. Doleys DM, Slapion MJ. The reduction of verbal repetitions by response cost controlled by a sibling. J Behav Ther Exp Psychiatry. 1975;6(1):61–3.

15. Douglas SN, Kammes R, Nordquist E, D’Agostino S. A pilot study to teach siblings to support children with complex communication needs. Commun Disord Q. 2018 Feb;39(2):346–55.

16. Dyson LL. A support program for siblings of children with disabilities: What siblings learn and what they like. Psychol Sch. 1998;35(1):57–65.

17. Evans J, Jones J, Mansell I. Supporting siblings: Evaluation of support groups for brothers and sisters of children with learning disabilities and challenging behaviour. J Learn Disabil. 2001;5(1):69–78.

18. Ferraioli SJ, Harris SL. Teaching joint attention to children with autism through a sibling-mediated behavioral intervention. Behav Interv. 2011 Nov;26(4):261–81.

19. American Psychiatric Association. Diagnostic and statistical manual of mental disorders. 4th ed. Washington, DC: American Psychiatric Association; 2000.

20. Fjermestad K, Pat P, Dearozet S, Vatne T, Hafting M, Jegannathan B. Manual-Based Group Intervention for Siblings and Parents of Children with Neurodevelopmental Disorders in Cambodia. J Dev Phys Disabil. 2021;33(5):839–56.

21. Fjermestad KW, Silverman WK, Vatne TM. Group intervention for siblings and parents of children with chronic disorders (SIBS-RCT): study protocol for a randomized controlled trial. Trials. 2020;21(1):1–12.

22. Gettings S, Franco F, Santosh PJ. Facilitating support groups for siblings of children with neurodevelopmental disorders using audio-conferencing: A longitudinal feasibility study. Child Adolesc Psychiatry Ment Health. 2015;9(1):1-15.

23. Granat T, Nordgren I, Rein G, Sonnander K. Group intervention for siblings of children with disabilities: a pilot study in a clinical setting. Disabil Rehabil. 2012;34(1):69–75.

24. Hancock TB, Kaiser AP. Siblings’ use of milieu teaching at home. Topics Early Child Spec Educ. 1996;16(2):168–90.

25. Hayden NK, McCaffrey M, Fraser-Lim C, Hastings RP. Supporting siblings of children with a special educational need or disability: An evaluation of Sibs Talk, a one-to-one intervention delivered by staff in mainstream schools. Support Learn. 2019;34(4):404–20.

26. James SD, Egel AL. A direct prompting strategy for increasing reciprocal interactions between handicapped and nonhandicapped siblings. J Appl Behav Anal. 1986;19(2):173–86.

27. Jones EA, Fiani T, Stewart JL, Neil N, McHugh S, Fienup DM. Randomized controlled trial of a sibling support group: Mental health outcomes for siblings of children with autism. Autism. 2020;24(6):1468–81.

28. Kryzak LA, Cengher M, Feeley KM, Fienup DM, Jones EA. A community support program for children with autism and their typically developing siblings: Initial investigation. J Intellect Disabil. 2015;19(2):159–77.

29. Kryzak LA, Jones EA. Sibling self-management: Programming for generalization to improve interactions between typically developing siblings and children with autism spectrum disorders. Dev Neurorehabil. 2017;20(8):525–37.

30. Lewandowski JF, Hutchins TL, Prelock PA, Murray-Close D. Examining the benefit of including a sibling in story-based intervention with a child with Asperger Syndrome. Contemp Issues Commun Sci Disord. 2014;41(Fall):179–95.

31. Lobato D, Tlaker A. Sibling intervention with a retarded child. Educ Treat Child. 1985;8(3):221–8.

32. Lobato D. Brief report: Preschool siblings of handicapped children - Impact of peer support and training. J Autism Dev Disord. 1985;15(3):345–50.

33. Lobato DJ, Kao BT. Integrated sibling-parent group intervention to improve sibling knowledge and adjustment to chronic illness and disability. J Pediatr Psychol. 2002;27(8):711–6.

34. Lobato DJ, Kao BT. Brief report: Family-based group intervention for young siblings of children with chronic illness and developmental disability. J Pediatr Psychol. 2005;30(8):678–82.

35. McCullough K, Simon SR. Feeling heard: a support group for siblings of children with developmental disabilities. Soc Work Groups. 2011;34(3–4):320–9.

36. McLinden SE, Miller LM, Deprey JM. Effects of a support group for siblings of children with special needs. Psychol Sch. 1991;28:230–7.

37. Miller NB, Cantwell DP. Siblings as therapists: a behavioral approach. Am J Psychiatry. 1976;133(4):447–50.

38. Neff ER, Betz AM, Saini V, Henry E. Using video modeling to teach siblings of children with autism how to prompt and reinforce appropriate play. Behav Interv. 2017;32(3):193–205.

39. Oppenheim-Leaf ML, Leaf JB, Dozier C, Sheldon JB, Sherman JA. Teaching typically developing children to promote social play with their siblings with autism. Res Autism Spectr Disord. 2012;6(2):777–91.

40. Özen A. Effectiveness of siblings-delivered iPad game activities in teaching social interaction skills to children with autism spectrum disorders. Kuram ve Uygulamada Egit Bilim. 2015;15(5):1287–303.

41. Phillips RSC. Intervention with siblings of children with developmental disabilities from economically disadvantaged families. Fam Soc. 1999;80(6):569–77.

42. Roberts RM, Ejova A, Giallo R, Strohm K, Lillie M, Fuss B. A controlled trial of the SibworkS group program for siblings of children with special needs. Res Dev Disabil. 2015;43:21–31.

43. Roberts RM, Ejova A, Giallo R, Strohm K, Lillie ME. Support group programme for siblings of children with special needs: predictors of improved emotional and behavioural functioning. Disabil Rehabil. 2016 Oct 15;38(21):2063–72.

44. Rye K, Hicks S, Falconer C. Evaluating a group for young people who have a sibling with a disability. Learn Disabil Pract. 2018 May 15;1.

45. Schreibman L, O’Neill RE, Koegel RL. Behavioral training for siblings of autistic children. J Appl Behav Anal. 1983;16(2):129–38.

46. Sheikh R, Patino V, Cengher M, Fiani T, Jones EA. Augmenting sibling support with parent-sibling training in families of children with autism. Dev Neurorehabil. 2019;22(8):542–52.

47. Smith T, Perry A. A sibling support group for brothers and sisters of children with autism. J Dev Disabil. 2004;11(1):77–88.

48. Spector V, Charlop MH. A sibling-mediated intervention for children with autism spectrum disorder: Using the Natural Language Paradigm (NLP). J Autism Dev Disord. 2018;48(5):1508–22.

49. American Psychiatric Association. Diagnostic and statistical manual of mental disorders. 5th ed. Arlington, VA: American Psychiatric Publishing, Inc.; 2013.

50. Stewart DA, Benson GT, Lindsey JD. A unit plan for siblings of handicapped children. Teach Except Child. 1987;19(3):24–8.

51. Stewart KK, Carr JE, LeBlanc LA. Evaluation of family-implemented behavioral skills training for teaching social skills to a child with Asperger’s disorder. Clin Case Stud. 2007;6(3):252–62.

52. Swenson-Pierce A, Kohl FL, Egel AL. Siblings as home trainers: A strategy for teaching domestic skills to children. J Assoc Pers with Sev Handicap. 1987;12(1):53–60.

53. Trent JA, Kaiser AP, Wolery M. The use of responsive interaction strategies by siblings. Topics Early Child Spec Educ. 2005;25(2):107–18.

54. Trent-Stainbrook A, Kaiser AP, Frey JR. Older siblings’ use of responsive interaction strategies and effects on their younger siblings with down syndrome. J Early Interv. 2007;29(4):273–86.

55. Tsao LL, Odom SL. Sibling-mediated social interaction intervention for young children with autism. Topics Early Child Spec Educ. 2006;26(2):106–23.

56. Tsao LL, McCabe H. Why won’t he play with me?: Facilitating sibling interactions. Young Except Child. 2010;13(4):24–35.

57. Tsao LL. Brothers as playmates for their siblings with developmental disabilities: A multiple-baseline design study. Child Youth Care Forum. 2020;49(3):409–30.

58. Walton KM, Ingersoll BR. Evaluation of a sibling-mediated imitation intervention for young children with autism. J Posit Behav Interv. 2012;14(4):241–53.

59. Lord C, Risi S, Lambrecht L, Cook, Jr. EH, Leventhal BL, DiLavore PC, et al. The Autism Diagnostic Observation Schedule—Generic: A Standard Measure of Social and Communication Deficits Associated with the Spectrum of Autism. J Autism Dev Disord. 2000;30(3):205–23.

60. Weinrott MR. A training program in behavior modification for siblings of the retarded. Am J Orthopsychiatry. 1974;44(3):362–75.

61. Williams PD, Hanson S, Karlin R, Ridder L, Liebergen A, Olson J, et al. Outcomes of a nursing intervention for siblings of chronically ill children: A pilot study. J Soc Pediatr Nurses. 1997;2(3):127–37.

62. Williams PD, Williams AR, Graff JC, Hanson S, Stanton A, Hafeman C, et al. A community-based intervention for siblings and parents of children with chronic illness or disability: The ISEE study. J Pediatr. 2003;143(3):386–93.

63. Canadian Institutes of Health Research. What is gender? What is sex? [Internet]. 2020 [cited 2021 Dec 12]. Available from: https://cihr-irsc.gc.ca/e/48642.html
