# Supplementary material for: Programs to Prepare Siblings for Future Roles to Support Their Brother or Sister with a Neurodevelopmental Disability: a Scoping Review
Source: Curr Dev Disord Rep. 2023 Feb 21;10(1):47–79. doi: 10.1007/s40474-023-00272-w (PMC9942034; doi:10.1007/s40474-023-00272-w)
Supplement: Supplementary file 3 — Supplementary file3 (DOCX 127 KB) [file 40474_2023_272_MOESM3_ESM.docx]

**Supplementary File 3.** Characteristics of programs on knowledge acquisition and skill development for the siblings.

| **Study and Program Name** | **Objective(s) for siblings without NDD** | **Duration, frequency, and context** | **Activities** | **Developers** | **Resources for Development** | **Facilitators** | **Conducted Evaluation? (Yes/No)** |
| --- | --- | --- | --- | --- | --- | --- | --- |
| Lobato 1985 [1] | To improve the participants' understanding of developmental disabilities; to increase their recognition of the strengths of themselves, of their sibling with a disability, and of other family members; and to improve their skills in constructively expressing negative emotions associated with their unique situations. | 1.5 hours once each week for 6 consecutive weeks at a children’s playroom at a clinic. | A combination of training procedures typically employed in behavioral social skills training for preschool-aged children, including modeling, coaching, rehearsal, role-play, and differential feedback. Other children were encouraged to act as models and to provide specific praise and feedback to one another. Puppets, human-figure dolls, art materials, and children's literature on disabilities were used throughout the workshop to structure activities and guide discussion. | Not listed. | Not listed. | Not listed. | No |
| Stewart et al. 1987 [2] | The overall goal of these sessions is to provide siblings with the experiences they need to develop a perspective that portrays their handicapped brothers and sisters in a positive light. | An example of a model unit plan illustrates 45-minute sessions for 5 days. | Guest speakers, lectures, discussions, role playing, field trips, media presentations, printer materials (e.g., novels by or about individuals with disabilities, newspaper and magazine articles, songs written by exceptional individuals, commercially developed curricula, books and pamphlets), paper and pencil tasks (e.g., word puzzles, essays, research papers, poems, drawings, etc.), and picnics. | Not listed. | Not listed. | Suggestions were provided for facilitators of each activity. | No |
| Crouthamel 1988 [3] | To provide support to siblings. | Eight consecutive Saturdays at a regional medical center. | Introductory session for children and parents to meet. Second session to view a videotaped discussion by a group of adult siblings of individuals with disabilities. Third session to ask medical questions to a nurse practitioner, with the use of medical books with illustrations and a model of the human brain. Remaining sessions for open conversations and planning for a newsletter that was an idea from a participant and endorsed by all participants. Final session to have a party with all family members. | Not listed. | Not listed. | A social worker and a nurse practitioner (also the sibling of a young adult with a developmental disability). | No |
| McLinden et al. 1991 [4] | To provide peer support, a forum for the expression of feelings (both positive and negative), and coping strategies for living with a sibling who has a disability | 1 hour per week for 6 weeks | The group focused on developing participants’ acceptance of both negative and positive feelings about their siblings. Information was provided about a wide range of disabilities. Numerous activities were utilized to provide group participants with a range of strategies to deal with peers and peer reactions to their siblings with disabilities, including homework assignments to reinforce concepts taught during formal group sessions. | Not listed. | Procedures from the literature. | Two state-certified school psychologists. | Yes |
| Williams et al. 1997 [5]  Intervention for Siblings: Experience Enhancement (ISEE) | To provide education, psychosocial and social-recreational components. | 5-6 hours for the educational component, 3-4 hours for the psychosocial component, and 1.5 hours for the social and recreational component. Average number of hours spent on the sessions was nine at a medical center. | The sibling intervention consisted of structured, educational, and psychosocial, and social and recreational group sessions with siblings of children with chronic illness. A discussion session was also held with parents about issues related to sibling adjustment. | Professional expertise of each clinical nurse specialist member of the study. | Not listed. | Educational component was conducted the clinical nurse specialists of each diagnostic condition and a graduate student in pediatric nursing. Psychosocial component was conducted by experienced facilitators on the study team. | Yes |
| Dyson 1998 [6] | To provide support to siblings of a sibling with NDD. | 2-hour sessions for six Saturday afternoon workshops at a community recreation center. | Arts and crafts, learning about disabilities, group discussions and sharing of sibling experiences, and recreational and social times. | Not listed. | Not listed. | Not listed. | Yes |
| Phillips 1999 [7] | To alleviate the stress caused by having a sibling with a disability by providing information about, and facilitating understanding of, developmental disabilities and by creating a context that provided social support from peers and adults. | 2.5 hours for 15 weeks at a community center. | Group discussions and check-ins, with a different topic relevant to siblings of an individual with a disability for each week. A variety of structured and unstructured recreational activities including computer games, videos, dance contests, theater arts, sports, gardening, and crafts. Each session included homework and tutoring. | Not listed. | Not listed. | Six team leaders who were paid community-center staff and seven volunteers who included community residents, high school students, and community center staff from other programs. | No |
| Evans et al. 2001 [8]  Facing the Challenge | Facing the Challenge  To assist siblings in developing positive strategies for living with their siblings who have learning disabilities and an associated challenging behaviour. | Three consecutive full-day sessions full days followed by six weekly evening sessions. Assessments were conducted in the participants' own home and during the group sessions. | Flashcards and video for siblings to learn how to explain their sibling's learning disabilities. Role-play scenarios were also used to explore the type of behaviours from their siblings with a disability and discuss how to manage these situations. Puppets and masks for how children visualized their sibling with a disability. Relaxation techniques were also taught, and the siblings practised games they could play with their siblings. Time was set aside each week to discuss with each sibling about goals of the session, as well as explore their input and strengths for the group to incorporate for the following session. | Not listed. | Not listed. | Four staff who included community nurses, outreach nurses and a psychologist. | Yes |
| Lobato et al. 2002 [9]  SibLink | To improve sibling knowledge, sibling adjustment to a chronic illness/developmental disability, and siblings' sense of connectedness to other children in similar family circumstances. | Six 90-minute group sessions conducted over a 6-8 week period | Two sessions targeted improving sibling knowledge and family information exchange. Two sessions targeted identifying and managing sibling emotions with problem-solving around challenging situations. One session focused on balancing the siblings' individual needs. The final session provided a review and graduation ceremony. Some activities to integrate the sibling and parent groups to enhance mutual understanding and perspective taking. For example, siblings created a video about their experiences that parents reviewed. | Study authors. | Based on existing literature and original activities created by the authors. | Two doctoral level trainees in psychology or psychiatry. | Yes |
| Smith et al. 2004 [10] | To increase knowledge and understanding of autism and related developmental disorders, provide the opportunity for siblings to discuss their feelings in an accepting atmosphere, help siblings to share ways of coping with difficult situations unique to having a sibling with autism (e.g., through role playing); enhance siblings' self-concepts, and encourage siblings to have fun in a supportive environment. | Weekly sessions for eight consecutive weeks at a health center. | Information sessions on autism and related disorders and discussions relating to feelings and attitudes associated with living with a sister with a developmental disability. Exercises, games, and activities also focused on fun and promotion of group cohesion. | Not listed. | Not listed. | Not listed. | Yes |
| Williams et al. 2004 [11] | To provide structured teaching about the sibling's illness, psychosocial sessions, a 5-day residential summer camp, and two booster sibling sessions. | 2-2.5 hour sessions at a 5-day residential camp with booster sessions at a medical center. | Educational sessions focused on medical information about the specific illness or disability. Psychosocial sessions focused on psychosocial issues, and siblings were encouraged to ask questions, share experiences, and talk about feelings of their sibling with a disability. Stress and ways of coping were discussed, and group games were played. Parent session focused on information exchange with nurse clinicians and other parents to enhance awareness of sibling needs. Booster sessions were provided to parents and siblings. | Consultation with pediatric nurse clinicians, nurse researchers, physicians, and allied health professional. | Based upon a review of the literature and consultations. | Pediatric nurse clinicians | No |
| D'Arcy et al. 2005 [12]  Sibshops | To provide opportunities to meet other siblings, to discuss common joys and concerns, to learn how to handle situations commonly experienced by siblings, and to learn about the implications of the needs of siblings with a disability. | 3-hour monthly sessions on Saturdays for four consecutive months. | Monthly meetings, where a group of siblings come together to share information about their siblings with disabilities and to have fun. The morning consists of high and low energy activities, interspersed with discussion about disability and each sibling's experience. | Not listed. | Based on a previously developed model. | Not listed. | Yes |
| Lobato et al. 2005 [13]  SibLink | To address sibling challenges. | Six 90-minute sessions of collateral and integrated sibling-parent groups. | The collateral sibling group activities alternated between explicitly focused on "main events" and other more social-recreational activities that implicitly addressed sibling connectedness. Two sessions targeted improving sibling knowledge and family discussions about the child's condition. Two sessions targeted identifying and managing sibling emotions with problem-solving around challenging situations typical for young siblings and one session focused on identifying the strengths of siblings and balancing the siblings' individual needs within the family. The final session provided a review and graduation ceremony. Integrated sibling and parent groups also were conducted to enhance mutual understanding and perspective taking. Siblings created a videotape about their experiences as a brother or sister that parents reviewed. Sibling and parent groups joined for some sessions to read books about siblings and illness or disability together, to engage in interactive exercises, and a graduation. | Not listed. | Not listed. | Two doctoral level psychology trainees. | Yes |
| McCullough et al. 2011 [14] | To provide a fun and stimulating environment in which siblings could relax and develop relationships with peers, to provide didactic information about developmental disabilities and psychosocial information about tools for coping with a special needs sibling, and to provide support for enhancing the development of a healthy self-concept and sense of efficacy. | Not listed. | Activities celebrated the special traits of participants and their siblings with NDD to underscore positive feelings of loyalty, self-efficacy, and self-esteem and to ensure that opportunities to express negative or ambivalent feelings were balanced with opportunities to find a positive perspective. | Not listed. | Strengths perspective was kept in mind. | Not listed. | No |
| Granat et al. 2012 [15] | To increase knowledge of the Sibling about their sibling's disability and improve the ability of the Sibling to hand their environment (e.g., answering questions from peers). To instruct siblings how to use problem-solving strategies in order to provide them with tools for dealing with their sibling relationship more effectively. | Two-hour sessions for six weeks. | Session focused on providing information about a particular disability, with questions, discussions, games. The sessions also included structured role-playing of problem-solving strategies. The Siblings compiled a personal notebook comprised of lecture notes, stories, and photos of role-playing activities. Practical exercises, social interactions, and games were included. The final session was a festive occasion that included a presentation of diplomas and the siblings' evaluations. | Not listed. | Based on previous research about sibling programs. | Clinical staff from an outpatient habilitation centre. | Yes |
| Gettings et al. 2015 [16] | To provide support to siblings, including group cohesiveness, installation of hope, psycho-education, and sharing of experiences. | One-hour sessions for eight consecutive weeks and were followed up three to six months after the last session. Sessions were in-person at a hospital school room and through audio-conferencing. | First two sessions focused on ice breaker exercises to encourage group cohesion. Session three provided Siblings with the opportunity to gain a better understanding of their siblings' diagnoses with the opportunity to ask questions. Session four focused on matters relating to school to enable siblings to share their experiences at school including talking about friendships. The fifth session allowed siblings to share stories about recreational activities and daily family life. Session six focused on problem-solving to enhance coping mechanisms for Siblings. The last session focused on the Siblings' thoughts about the future including their hopes for their sibling with a disability and their dreams. | Not listed. | Adaptation from existing models. | A child psychiatrist and a clinical nurse specialist, who are both experienced clinicians. | Yes |
| Kryzak et al. 2015 [17]  Support and Skills Program | To decrease typical sibling maladjustment, increase sibling's knowledge about autism spectrum disorder, increase sibling social network, and improve sibling interactions. | 2-hour sessions on seven Saturdays over a 9-week period in the fall and over a 9-week period in the spring at two university settings. | Siblings with autism spectrum disorder received individualized intervention in social, communication, and play/leisure skills, while the Siblings participated in a support group focused on developing a network of peers who face similar family challenges, learning about autism spectrum disorder, and learning coping strategies. Then the sibling with autism spectrum disorder and their siblings attended an inclusive recreation time where they practiced what they had learned in their separate groups. Each week covered a specific topic for sessions for the Siblings. In the first few weeks, topics focused on learning about other group members, while the latter weeks focused on knowledge about autism spectrum disorder. Each weekly meeting began with an icebreaker game and ended with a homework assignment. This assignment helped inform parents of the activities in the support group and encouraged siblings to engage in activities with their sibling with autism spectrum disorder at home. | First and last authors. | Not listed. | Licensed and credentialed professionals with experience with autism spectrum disorder treatment with student volunteers. | No |
| Roberts et al. 2015 [18]  SibworkS | To get to know each other and establish group goals, to note similarities and differences between oneself and one's sibling, as well as sharing knowledge about various disabilities, to express feelings and seek social support, to use problem-solving approaches, to develop strategies for coping with stresses, including those relating to one's sibling, and to recognize one's individuality, even in terms of what one has taken away from the program. | 2-hour sessions for 6 weeks. | Activities related to the objectives of each session. | Not listed. | Based on cognitive-behavioural therapy principles. | A paid group leader, who was a postgraduate clinical psychology student and a Provisionally Registered Psychologist supervised by a Clinical Psychologist. The group leader was assisted by a volunteer who had experience working with children, and, in most cases, experience in supervising outings for the siblings of children with disabilities. | Yes |
| Roberts et al. 2016 [19]  SibworkS | To get to know other participants and establish group goals, to recognize similarities and differences between oneself and one's sibling with special needs while sharing knowledge about different disabilities, to express feelings and seek social support, to apply problem-solving approaches, to develop strategies for coping with stresses, including those relating to one's sibling with special needs, and to recognize one's individuality, even in terms of what one has taken away from the program. | Weekly 2-hour sessions for 6 weeks. | Activities matched the intervention objectives and based on previous work that was found to be acceptable to siblings and/or families, as well as promote well-being (e.g., building the Sibling's knowledge of disability, developing coping strategies). | Not listed. | Based on a model of family stress and adaptation. | Paid group leader, who was a postgraduate clinical psychology student and a provisionally registered psychologist. The group leader was assisted by a volunteer who had experience working with children and, in most cases, experience in the supervision of outings for the siblings of children with special needs. Additionally, a research assistant attended all sessions in an administrative capacity | No |
| Brouzos et al. 2017 [20] | To provide psycho-education about autism, emotional education, cognitive restructuring, training in relaxation techniques, problem-solving and social skills training and psycho-education about self-acceptance. | 90-minute sessions for 8 weeks on consecutive Saturdays at an activity center for children with disabilities. | Weekly meetings, education and structured activities. Each group session consisted of four parts. In the first part, the introduction of the session's topic was followed by a discussion among the members and the leader. In the second part, the leader introduced the structured activities that the Siblings often carried out individually or in pairs. In the third part the activity was processed. The final part included the closing of the session. | Study authors. | Not listed. | Female Master's student in a Counselling Psychology program, who had attended a postgraduate level group counseling course. She had an educational background in primary education and experience as a tutor for children with autism spectrum disorder. | No |
| Rye et al. 2018 [21] | To increase siblings’ understanding about disabilities, provide a space for peer support and help young people learn skills to help themselves and their siblings at difficult times. | Two-hour sessions for ten weeks at the Ealing Service for Children with Additional Needs multiagency building. | Icebreakers for siblings to get to know one another. Discussion-based activities on disability to increase knowledge and understanding. Discussion-based activities on feelings to develop skills in recognising their own and others’ feelings. Problem-solving activities to encourage development of coping strategies. Arts and crafts and team building games to encourage young people to work together. A final celebratory session which included a fun activity chosen by the young people. | Not listed. | Based on the structure recommended by Sibs, a national charity for people with a sibling with a disability. | The facilitators were two assistant psychologists who also received sibling group leader training from the organization. | No |
| Hayden et al. 2019 [22]  Sibs Talk | To improve siblings' well-being and their engagement with learning | 25-35 minute sessions for ten sessions during a school term at school. | Siblings were guided through an activity page in a booklet. The first session started with sharing basic information about each sibling's family and circumstances to help the sibling develop trust and rapport with the staff member leading the intervention. As the booklet progressed, the sessions focus in more depth on their brother or sisters' disability or condition, the Siblings' feelings and experiences, and the issues that were challenging for them at home and school. The sessions also looked at the skills, knowledge and attributes that Siblings have acquired and how their school can support them. | Sibs, a charity in the United Kingdom | Not listed. | Staff members who attend a two-hour training session at their own school or at a host school nearby. The training focused on the micro skills required for listening to and acknowledging siblings' feelings. Staff could contact Sibs staff for further advice if required. | Yes |
| Sheikh et al. 2019 [23] | The sibling support program focused on learning about autism spectrum disorder, sharing feelings, learning coping skills, problem solving, advocacy, and creating a peer support network. The parent-sibling training focused on increasing prosocial behaviour directed toward the sibling with autism spectrum disorder. | 1.5 hour support group session for ten weeks with 30-45 minute parent-sibling training for three to six weeks at a university. | For the support group, games and activities related to a specific topic and goal. Each session began with some warm-up games while the group waited for all the siblings to arrive. Then the leader introduced the main activity for that week, spending the majority of the time on that activity, followed by a short snack. During the first few weeks, activities and discussions focused on learning about other group members to develop group rapport and a peer network and learn about group members’ similarities and differences and begin to share their feelings about their siblings with autism spectrum disorder. Siblings took home weekly challenges to practice something at home or talk to their parents .This helped inform parents of the activities in the support group and encouraged siblings to engage in activities with their sibling with autism spectrum disorder at home. Immediately after a parent-intervention session, the parent, Sibling, and sibling with autism spectrum disorder participated in family play sessions. | Not listed. | Based on the curriculum of other sibling support groups. | First year graduate students pursuing a Master’s degree in Applied Behavior Analysis, each with over 2 years of experience working with children with autism spectrum disorder, and one student completing a Bachelor’s degree in Psychology. developmental disabilities and behavior therapists working with children with autism spectrum disorder and families. | No |
| Burke et al. 2020 [24]  Sibling Ambassadors Program | To increase awareness of the sibling experience and sibling needs among siblings of individuals with intellectual and developmental disabilities and the public more generally. | 15 hours for two days. | Education, in which siblings learned about disability policy, advocacy, and peer support. Training included: didactic instruction, small group activities, skill-building sessions, individual reflections, and time for siblings to socialize with one another. At the end of the training, participants created an action plan with targeted activities to increase awareness of the sibling experience in their communities. | The Sibling Leadership Network. | Based on literature. | Adult siblings of individuals with intellectual and developmental disabilities; these siblings were affiliated with the Sibling Leadership Network. | No |
| Fjermestad et al. 2020 [25]  SIBS intervention | To provide siblings with an opportunity to discuss the disorder of their brother/sister, and associated emotions and family challenges. | Five sessions in one day at a mental health clinic for children and adolescents. | Sessions 1, 2, and 4 are parallel (separate) group sessions for siblings and parents. Sessions 3 and 5 are integrated sibling-parent dialogues in which each sibling and parent talk together in pairs. Session 1 was an introduction. Sessions 2–3 focused on siblings’ disorder knowledge based on a semi-structured group interview. Sessions 4–5 focused on siblings' emotional experiences based on cognitive-behavioral principles about thoughts and behavior influence emotions. In Sessions 2 and 4, siblings prepared lists of questions about the disorder and experienced family challenges, respectively, which a group leader brings to the parent group for discussion. In Sessions 3 and 5, each sibling discussed questions and challenges with their parent. | Not listed. | Not listed. | Group leaders were clinical staff (i.e., psychologists, social workers, psychiatrists, and art therapist) with training to present the intervention. | Yes |
| Fjermestad et al. 2020 [26]  SIBS intervention | To improve their mental health and well-being. | 5 sessions delivered over 2 days, 1 week apart. Day 1 comprised of sessions 1-3 (3.0 h including breaks). Day 2 comprised of sessions 4-5 (2.5 h including breaks) at municipal and specialist health service sites. | Sessions 1, 2, and 4 are parallel (separate) group sessions for siblings and parents. Sessions 3 and 5 are integrated sibling-parent dialogs in which each sibling and parent talk together, separate from other participants. Sessions primarily focused on disorder knowledge and emotional experiences, in relation to the overarching component, family communication. | Study authors developed the manual. | Not listed. | Group leaders with employment in municipal or specialist health services, health professional training, completion of a training package. All group leaders will be invited to supervision webinars 2-3 times per year. | No |
| Jones et al. 2020 [27] | To provide support to siblings, including a discussion of feelings related to the sibling with autism spectrum disorder and family, problem-solving and coping skills, peer network, or information about autism spectrum disorder. | 2-hour sessions for 10 Saturday or Sunday mornings. | During the first hour, siblings with autism spectrum disorder received individualized instruction in social, communication, and skills while siblings participated in either the support group or attention-only control group. During the second hour, all siblings had inclusive recreation activities (e.g., stretches, relay races). Lessons and activities focused on characteristics of autism spectrum disorder, different ways to cope with frustrations with siblings with autism spectrum disorder (e.g., take a deep breath, tell a parent) and who to go to for help. Siblings had weekly challenges to practice something at home or talk to their parents. This helped inform parents of the activities in the support group and encouraged siblings to engage in activities with their sibling with autism spectrum disorder at home. | Not listed. | Based on sibling programs previously described in the literature. | A graduate student with the assistance of another graduate or advanced undergraduate student (trained and supervised by the first and last authors), with a psychology background and experience implementing the program. | No |

Abbreviation: NDD, neurodevelopmental disabilities.

**References**

1. Lobato D. Brief report: Preschool siblings of handicapped children - Impact of peer support and training. J Autism Dev Disord. 1985;15:345–50.

2. Stewart DA, Benson GT, Lindsey JD. A unit plan for siblings of handicapped children. Teach Except Child. 1987;19:24–8.

3. Crouthamel CS. Siblings of handicapped children: A group support program. Early Child Dev Care. 1988;37:119–31.

4. McLinden SE, Miller LM, Deprey JM. Effects of a support group for siblings of children with special needs. Psychol Sch. 1991;28:230–7.

5. Williams PD, Hanson S, Karlin R, Ridder L, Liebergen A, Olson J, et al. Outcomes of a nursing intervention for siblings of chronically ill children: a pilot study. Journal of the Society of Pediatric Nurses. 1997;2:127–37.

6. Dyson LL. A support program for siblings of children with disabilities: What siblings learn and what they like. Psychol Sch. 1998;35:57–65.

7. Phillips RSC. Intervention with siblings of children with developmental disabilities from economically disadvantaged families. Fam Soc. 1999;80:569–77.

8. Evans J, Jones J, Mansell I. Supporting siblings: Evaluation of support groups for brothers and sisters of children with learning disabilities and challenging behaviour. J Learn Disabil. 2001;5:69–78.

9. Lobato DJ, Kao BT. Integrated sibling-parent group intervention to improve sibling knowledge and adjustment to chronic illness and disability. J Pediatr Psychol. 2002;27:711–6.

10. Smith T, Perry A. A sibling support group for brothers and sisters of children with autism. Journal on Developmental Disabilities. 2004;11:77–88.

11. Williams PD, Williams AR, Graff JC, Hanson S, Stanton A, Hafeman C, et al. A community-based intervention for siblings and parents of children with chronic illness or disability: The ISEE study. Journal of Pediatrics. 2003;143:386–93.

12. D’Arcy F, Flynn J, McCarthy Y, O’Connor C, Tierney E. Sibshops. An evaluation of an interagency model. Journal of Intellectual Disabilities. 2005;9:43–57.

13. Lobato DJ, Kao BT. Brief report: Family-based group intervention for young siblings of children with chronic illness and developmental disability. J Pediatr Psychol. 2005;30:678–82.

14. McCullough K, Simon SR. Feeling heard: a support group for siblings of children with developmental disabilities. Soc Work Groups. 2011;34:320–9.

15. Granat T, Nordgren I, Rein G, Sonnander K. Group intervention for siblings of children with disabilities: a pilot study in a clinical setting. Disabil Rehabil. 2012;34:69–75.

16. Gettings S, Franco F, Santosh PJ. Facilitating support groups for siblings of children with neurodevelopmental disorders using audio-conferencing: A longitudinal feasibility study. Child Adolesc Psychiatry Ment Health. 2015;9(1): 1-15.

17. Kryzak LA, Cengher M, Feeley KM, Fienup DM, Jones EA. A community support program for children with autism and their typically developing siblings: Initial investigation. Journal of Intellectual Disabilities. 2015;19:159–77.

18. Roberts RM, Ejova A, Giallo R, Strohm K, Lillie M, Fuss B. A controlled trial of the SibworkS group program for siblings of children with special needs. Res Dev Disabil. 2015;43:21–31.

19. Roberts RM, Ejova A, Giallo R, Strohm K, Lillie ME. Support group programme for siblings of children with special needs: predictors of improved emotional and behavioural functioning. Disabil Rehabil. 2016;38:2063–72.

20. Brouzos A, Vassilopoulos SP, Tassi C. A psychoeducational group intervention for siblings of children with autism spectrum disorder. Journal for Specialists in Group Work. 2017;42:274–98.

21. Rye K, Hicks S, Falconer C. Evaluating a group for young people who have a sibling with a disability. Learning Disability Practice. 2018;1.

22. Hayden NK, McCaffrey M, Fraser-Lim C, Hastings RP. Supporting siblings of children with a special educational need or disability: An evaluation of Sibs Talk, a one-to-one intervention delivered by staff in mainstream schools. Support for Learning. 2019;34:404–20.

23. Sheikh R, Patino V, Cengher M, Fiani T, Jones EA. Augmenting sibling support with parent-sibling training in families of children with autism. Dev Neurorehabil. 2019;22:542–52.

24. Burke MM, Lee CE, Carlson SR, Arnold CK. Exploring the preliminary outcomes of a sibling leadership program for adult siblings of individuals with intellectual and developmental disabilities. Int J Dev Disabil. 2018;0:1–8.

25. Fjermestad K, Pat P, Dearozet S, Vatne T, Hafting M, Jegannathan B. Manual-Based Group Intervention for Siblings and Parents of Children with Neurodevelopmental Disorders in Cambodia. J Dev Phys Disabil. 2021;33:839–56.

26. Fjermestad KW, Silverman WK, Vatne TM. Group intervention for siblings and parents of children with chronic disorders (SIBS-RCT): study protocol for a randomized controlled trial. Trials. 2020;21:1–12.

27. Jones EA, Fiani T, Stewart JL, Neil N, McHugh S, Fienup DM. Randomized controlled trial of a sibling support group: Mental health outcomes for siblings of children with autism. Autism. 2020;24:1468–81.
