# Supplementary material for: Programs to Prepare Siblings for Future Roles to Support Their Brother or Sister with a Neurodevelopmental Disability: a Scoping Review
Source: Curr Dev Disord Rep. 2023 Feb 21;10(1):47–79. doi: 10.1007/s40474-023-00272-w (PMC9942034; doi:10.1007/s40474-023-00272-w)
Supplement: Supplementary file 4 — Supplementary file4 (DOCX 112 KB) [file 40474_2023_272_MOESM4_ESM.docx]

**Supplementary File 4.** Outcomes of programs focused on knowledge acquisition and skill development, and key findings for the siblings.

| **Study** | **Program Outcomes** | **Key findings for the siblings** |
| --- | --- | --- |
| Lobato 1985 [1] | Knowledge acquisition and skill development | Overall, participating in the workshop program led to positive changes in statements of self-descriptions and increased accuracy in describing disabilities. No negative side effects in behaviour. Parents reported satisfaction to have conversations with their child. |
| Stewart et al. 1987 [2] | Knowledge acquisition | Not applicable. |
| Crouthamel 1988 [3] | The author shared about the siblings' perspective from participating in the program | Enjoyment in meeting other siblings of individuals with disabilities and development of awareness of disabilities. |
| McLinden et al. 1991 [4] | Knowledge acquisition, skill development, and satisfaction with the program | Limited evidence of program effectiveness on knowledge, attitudes, self-concept, or problem behaviour. Reported enjoyment and higher levels of social support. Parents reported improvements in behaviour towards the sibling with disability. |
| Williams et al. 1997 [5] | Knowledge acquisition | A common theme found was that of siblings feeling physically and emotionally isolated from their parents, Thus, the results provide evidence that the family is a system of interrelated individuals, and that each member affects (and is affected by) other members of the system.    The study intervention showed positive effects on siblings of a sibling with a chronic health condition, based on the results of the knowledge test. In addition, the parents validated the usefulness of the program for the siblings in both their numerical rating of it and their verbatim descriptions of its specific benefits to siblings. |
| Dyson 1998 [6] | Knowledge acquisition | Significant increase in learning, such as ways to improve relationships with the sibling with NDD, and understanding about and empathy for people with disabilities. |
| Phillips 1999 [7] | Knowledge acquisition and social support. | Decrease in sibling-related stress, increased social support from peers and staff, less depression and anxiety, and higher self-esteem. |
| Evans et al. 2001 [8] | Satisfaction with the program, knowledge acquisition and skill development. | Development of self-esteem, knowledge about the needs of the sibling with NDD, improvement in relationships among family members, receptivity to supporting therapeutic intervention s at home. |
| Lobato et al. 2002 [9] | Knowledge acquisition and skill development. | Increased knowledge and sibling connectedness. Decreased behavioural problems from sibling reports, and decreased behavioural problems from parent reports. No negative emotional or behavioural outcomes reported. |
| Smith et al. 2004 [10] | Knowledge acquisition and skill development | Greater knowledge of autism spectrum disorder and better self-concepts (i.e., how they feel about themselves). Enjoyment of experiments reported from participating in the program. |
| Williams et al. 2004 [11] | Knowledge acquisition and skill development | Improvements in outcomes of sibling mood, attitudes toward illness and toward the sibling with NDD, social support, and self-esteem, as well as decreases in reported sibling behaviour problems. |
| D'Arcy et al. 2005 [12] | Knowledge acquisition and sharing of experiences. | Achievement of program goals. 81% of siblings wish to meet again after the program. Reported enjoyment, consistent attendance rates, able to articulate difficulties and challenging situations. Opportunity to learn something new. Ability to describe disability in terms of a social model. Program benefits were observed by siblings and parents. |
| Lobato et al. 2005 [13] | Knowledge acquisition and skill development | Increased knowledge of disability, sense of connectedness, and perceptions of self-competence. No negative emotional or behavioural outcomes observed. |
| McCullough et al. 2011 [14] | Although not measured, the goals of the program were on knowledge acquisition and skill development. | The group modality provided a rich resource for siblings of children with developmental disabilities. |
| Granat et al. 2012 [15] | Knowledge acquisition and skill development (i.e., problem-solving skills). | Ability to explain the NDD of their sibling increased. Less time spend with their sibling with NDD, but had more fun when together. For siblings of a sibling with autism spectrum disorder, there were no significant changes in problem-solving strategies but there was increased admiration for their sibling. For siblings of a sibling with intellectual disabilities, there was an increase in the subscale Dominance, but also greater affection for their sibling. |
| Gettings et al. 2015 [16] | Knowledge acquisition | Provided descriptions of behavioural difficulties of their sibling with NDD that was consistent with parent reports. Increased number and range of people siblings spoke to about their concerns, for example, another sibling support group member. Increased feelings of preparation due to a better understanding of the NDD. |
| Kryzak et al. 2015 [17] | Knowledge acquisition and skill developing (i.e., coping strategies) | Significant improvements in the siblings' adjustment and peer network as well as some changes in knowledge about autism spectrum disorder and interactions with the sibling with autism spectrum disorder. Decreases in depression, anxiety, negative self-esteem, and interpersonal problems. |
| Roberts et al. 2015 [18] | Knowledge acquisition, skill development, satisfaction with the program | Improved emotional and behavioral functioning immediately after the program and maintained after 3 months. Enhanced self-esteem and less avoidant coping, but was not maintained at follow-up. |
| Roberts et al. 2016 [19] | Knowledge acquisition and skill development | Greater reduction in emotional and behavioural problems if the siblings had greater emotional and behavioural problems at baseline and were from families where the sibling with NDD had more severe symptoms, which were maintained at 3 months post-intervention. |
| Brouzos et al. 2017 [20] | Knowledge acquisition, skill development | Significant increase in understanding about autism spectrum disorder. Reduction of adjustment difficulties and emotional/behavioural problems. Younger participants were more likely to acquire valid information about autism spectrum disorder, whereas older participants were more likely to experience a decrease in their coping/adjustment difficulties. |
| Rye et al. 2018 [21] | Knowledge acquisition and skill development | Five themes identified: meeting similar people, a break from home, enjoyment of the activities-led group, building confidence and self-esteem, and learning and applying knowledge about disabilities. The sibling group offered young people a chance to meet with others in a similar position and for some this was the first time they had knowingly done so. The program also served to increase knowledge about disabilities and teach coping strategies the young people could use at difficult times, and some felt it did equip them with more knowledge about their sibling’s disability. |
| Hayden et al. 2019 [22] | Knowledge acquisition and skill development | Improvements on the Strengths and Difficulties Questionnaire that measures behavioural and emotional well-being, specifically there were statistically significant changes on hyperactivity scores, prosocial behaviours, and emotional problems. Some changes in how siblings feel about school, conduct problems, and peer problems but were not statistically significant. Siblings indicated that they learned new things about disability more generally. They could name individuals who they could talk to about sibling issues. Mostly positive comments about sibling relationships, although there were a few negative comments such as experiences of physical aggression from their sibling with a disability. |
| Sheikh et al. 2019 [23] | Knowledge acquisition and skill development | Increase in prosocial behaviours, and one sibling reported improvements in the sibling relationship. |
| Burke et al. 2020 [24] | Participation in training activities, community or political empowerment, advocacy, motivation to impact change, connectedness to the disability field | The program positively correlated with the intended outcomes of connectedness and empowerment, although these outcomes were not maintained at follow-up. Siblings developed solidarity with other siblings, felt empowered, and learned about new resources. |
| Fjermestad et al. 2020 [25] | Knowledge acquisition and satisfaction with the program. | Self-reported mental health remained unchanged. High satisfaction from siblings and parents. |
| Fjermestad et al. 2020 [26] | Knowledge acquisition | Not applicable. |
| Jones et al. 2020 [27] | Knowledge acquisition and skill development (i.e., problem-solving and coping skills) | Significant improvements in externalizing behaviour and coping skills when compared to the control group. When the siblings with autism spectrum disorder had more severe symptoms, the program appeared to provide a buffer again symptoms of anxiety and depression for the siblings. No changes in support network. Older siblings were found to be helpful during discussions with the younger siblings. |

Abbreviation: NDD, neurodevelopmental disabilities.

**References**

1. Lobato D. Brief report: Preschool siblings of handicapped children - Impact of peer support and training. J Autism Dev Disord. 1985;15:345–50.

2. Stewart DA, Benson GT, Lindsey JD. A unit plan for siblings of handicapped children. Teach Except Child. 1987;19:24–8.

3. Crouthamel CS. Siblings of handicapped children: A group support program. Early Child Dev Care. 1988;37:119–31.

4. McLinden SE, Miller LM, Deprey JM. Effects of a support group for siblings of children with special needs. Psychol Sch. 1991;28:230–7.

5. Williams PD, Hanson S, Karlin R, Ridder L, Liebergen A, Olson J, et al. Outcomes of a nursing intervention for siblings of chronically ill children: a pilot study. Journal of the Society of Pediatric Nurses. United States; 1997;2:127–37.

6. Dyson LL. A support program for siblings of children with disabilities: What siblings learn and what they like. Psychol Sch. 1998;35:57–65.

7. Phillips RSC. Intervention with siblings of children with developmental disabilities from economically disadvantaged families. Families in Society. 1999;80:569–77.

8. Evans J, Jones J, Mansell I. Supporting siblings: Evaluation of support groups for brothers and sisters of children with learning disabilities and challenging behaviour. J Learn Disabil. 2001;5:69–78.

9. Lobato DJ, Kao BT. Integrated sibling-parent group intervention to improve sibling knowledge and adjustment to chronic illness and disability. J Pediatr Psychol. 2002;27:711–6.

10. Smith T, Perry A. A sibling support group for brothers and sisters of children with autism. Journal on Developmental Disabilities. 2004;11:77–88.

11. Williams PD, Williams AR, Graff JC, Hanson S, Stanton A, Hafeman C, et al. A community-based intervention for siblings and parents of children with chronic illness or disability: The ISEE study. Journal of Pediatrics. 2003;143:386–93.

12. D’Arcy F, Flynn J, McCarthy Y, O’Connor C, Tierney E. Sibshops. An evaluation of an interagency model. Journal of Intellectual Disabilities. 2005;9:43–57.

13. Lobato DJ, Kao BT. Brief report: Family-based group intervention for young siblings of children with chronic illness and developmental disability. J Pediatr Psychol. 2005;30:678–82.

14. McCullough K, Simon SR. Feeling heard: a support group for siblings of children with developmental disabilities. Soc Work Groups. 2011;34:320–9.

15. Granat T, Nordgren I, Rein G, Sonnander K. Group intervention for siblings of children with disabilities: a pilot study in a clinical setting. Disabil Rehabil. 2012;34:69–75.

16. Gettings S, Franco F, Santosh PJ. Facilitating support groups for siblings of children with neurodevelopmental disorders using audio-conferencing: A longitudinal feasibility study. Child Adolesc Psychiatry Ment Health. 2015;9(1):1-15.

17. Kryzak LA, Cengher M, Feeley KM, Fienup DM, Jones EA. A community support program for children with autism and their typically developing siblings: Initial investigation. Journal of Intellectual Disabilities. 2015;19:159–77.

18. Roberts RM, Ejova A, Giallo R, Strohm K, Lillie M, Fuss B. A controlled trial of the SibworkS group program for siblings of children with special needs. Res Dev Disabil. 2015;43:21–31.

19. Roberts RM, Ejova A, Giallo R, Strohm K, Lillie ME. Support group programme for siblings of children with special needs: predictors of improved emotional and behavioural functioning. Disabil Rehabil 2016;38:2063–72.

20. Brouzos A, Vassilopoulos SP, Tassi C. A psychoeducational group intervention for siblings of children with autism spectrum disorder. Journal for Specialists in Group Work. 2017;42:274–98.

21. Rye K, Hicks S, Falconer C. Evaluating a group for young people who have a sibling with a disability. Learning Disability Practice. 2018;1.

22. Hayden NK, McCaffrey M, Fraser-Lim C, Hastings RP. Supporting siblings of children with a special educational need or disability: An evaluation of Sibs Talk, a one-to-one intervention delivered by staff in mainstream schools. Support for Learning. 2019;34:404–20.

23. Sheikh R, Patino V, Cengher M, Fiani T, Jones EA. Augmenting sibling support with parent-sibling training in families of children with autism. Dev Neurorehabil. 2019;22:542–52.

24. Burke MM, Lee CE, Carlson SR, Arnold CK. Exploring the preliminary outcomes of a sibling leadership program for adult siblings of individuals with intellectual and developmental disabilities. Int J Dev Disabil. 2018;0:1–8.

25. Fjermestad K, Pat P, Dearozet S, Vatne T, Hafting M, Jegannathan B. Manual-Based Group Intervention for Siblings and Parents of Children with Neurodevelopmental Disorders in Cambodia. J Dev Phys Disabil. 2021;33:839–56.

26. Fjermestad KW, Silverman WK, Vatne TM. Group intervention for siblings and parents of children with chronic disorders (SIBS-RCT): study protocol for a randomized controlled trial. Trials. 2020;21:1–12.

27. Jones EA, Fiani T, Stewart JL, Neil N, McHugh S, Fienup DM. Randomized controlled trial of a sibling support group: Mental health outcomes for siblings of children with autism. Autism. 2020;24:1468–81.
