# Supplementary material for: Programs to Prepare Siblings for Future Roles to Support Their Brother or Sister with a Neurodevelopmental Disability: a Scoping Review
Source: Curr Dev Disord Rep. 2023 Feb 21;10(1):47–79. doi: 10.1007/s40474-023-00272-w (PMC9942034; doi:10.1007/s40474-023-00272-w)
Supplement: Supplementary file 5 — Supplementary file5 (DOCX 141 KB) [file 40474_2023_272_MOESM5_ESM.docx]

**Supplementary File 5.** Characteristics of programs focused on empowerment by training siblings to teach skills to their sibling with neurodevelopmental disabilities.

| **Study and**  **Program Name** | **Objective(s) for siblings with NDD** | **Objective(s) for siblings without NDD** | **Duration, frequency, and context** | **Activities** | **Developers** | **Resources for Development** | **Facilitators** | **Conducted Evaluation? (Yes/No)** |
| --- | --- | --- | --- | --- | --- | --- | --- | --- |
| Weinrott et al. 1974 [1]  Sibling Training Program | Not listed. | To providing behavioral modification training strategies to siblings of a sibling with an intellectual disability. | 7 weeks at Camp Freedom. | On the first four days at camp, each sibling created their own schedule based on the guidelines from the program directors: each sibling would observe three activities (classes) of their choosing and attend two group meetings. On the third day, the emphasis shifted from observation to guided instruction and interaction with campers. siblings were no longer watching, but were teaching. Methods of dealing with resistance and aggression were also demonstrated. Siblings were given practice working with one or two campers under direct guidance consisting of specific instructions and prompts. Classes during the third day usually ended with the sibling working on a one-to-one basis with their camper sibling. Sessions included information about speech (e.g., developing expressive language), reading and readiness (e.g., how to conduct a teaching session at home), creative dramatics (e.g., play skills), and intellectual disabilities including medical considerations. There were daily swimming periods and recreational activity for which they would leave camp. These events included horseback riding, bowling, seeing a movie, or going out for a pizza. | Not listed. | Not listed. | Two graduate students. | Yes |
| Doleys et al. 1975 [2] | To reduce the number of verbal repetitions. | To implement a behavior modification program using a response cost contingency. | 2 weeks for intervention. | Not listed. | 19 year old sibling, a student in an undergraduate course in the experimental analysis of behaviour. | Not listed. | Not listed. | No |
| Miller et al. 1976 [3] | Case 1. To facilitate development of the sibling with NDD by shaping her speech and encouraging her to take risks and to play alone for as long as 45 minutes.    Case 2. One of the behaviour selected for intervention was to reduce the taking of food from the refrigerator for the sibling with NDD. | To train siblings to be involved in behavioural treatments for their sibling with NDD, and to increase positive interactions between siblings. | Not listed. | Case 1. The siblings were brought into the program; they observed the mother playing with Sally and she explained social learning principles to them in the therapist's presence.    Case 2. During the first two sessions, social learning principles were taught to the family through didactic materials and role playing. A schedule was made so that each sibling had 2 regular days "on call" during which he or she might have to help with the sibling with NDD. On other days they were free to plan their own activities. In addition, each sibling was asked to spend 15 minutes a day in a one-to-one activated related to the interests of the sibling with NDD, such as playing with cars or going for a walk. During these sessions they were to encourage eye contact, coherent speech, and appropriate social behaviors. | Not listed. | Not listed. | Therapist | No |
| Colletti et al. 1977 [4] | Not listed. | To train siblings as behaviour modification aides. | 20-minute sessions for six baseline sessions followed by six intervention sessions at home. | Experiment 1: The sibling was asked to carry out a cue from the experimenter, food reinforcement, and praise. The sibling was told to refrain from physical or further verbal interaction with her sister. For example, the sibling asked her sister with NDD to string the bead, receiving a cue to do so from the experimenter every 60 seconds.  Experiment 2: The siblings were asked to carry out a task with their sibling with NDD. When each sibling reached his first intervention phase, he was told that he would now be allowed to give the target child candy for doing the task correctly. The sibling with NDD was asked by the sibling to print letters on paper with crayon. | Not listed. | Not listed. | Not listed. | No |
| Schreibman et al. 1983 [5] | To learn a task or behaviour, and respond appropriately to prompts. | To provide siblings with a set of generalizable behavior modification skills, which they could use in a variety of settings with a variety of target behaviors. | Approximately 30 minutes for each session, a total of 8 sessions at home. | During the first training session the sibling and trainer viewed a videotape which presented examples of behavior therapy with autistic children. Reinforcement, shaping, chaining, and discrete trial techniques were discussed. Next, the trainer and sibling discussed how behavior modification procedures could be applied to everyday situations involving problem behaviors, using examples from a training manual. The next training step involved instruction to the siblings while they worked with their sibling with autism spectrum disorder. A specific target behavior was chosen and worked on for approximately 30 min. The trainer would periodically interrupt briefly with corrective or positive feedback on the sibling's progress. If the sibling had trouble applying a particular procedure, the trainer modeled the procedure and asked the sibling to try again until he or she could perform it correctly. | Not listed. | Based on literature. | Trainer. | No |
| Lobato et al. 1985 [6] | To acquire skills for improved functioning (i.e., self-care and domestic skills), specifically the goals selected were independent toothbrushing and bedmaking. | To train the sibling as a primary therapist for the sibling with NDD. | Weekly meetings for five consecutive weeks. Meetings every three weeks throughout the remainder of the project. | During the training sessions, the sibling participant was required to demonstrate mastery of the principles, application, and evaluation of behavior analysis using quizzes and workshops. As the final step in her training, the sibling developed a proposal of the instructional and evaluation methods she would use for the project. | Not listed. | Based on literature. | First author. | No |
| James et al. 1986 [7] | To increase reciprocal interactions between siblings. | To train siblings to increase interactions with their sibling with a disability. | Session ranged from 12-15 minutes.  Average of 5 days per week, no more than two training sessions on a single day, with 4-6 hours separating any two sessions. Total number of sessions ranged from 4-8 at home. | Modeling session to demonstrate how to initiate interactions, prompt responses, and reinforce both initiations and responses. The siblings practiced these skills with feedback and prompts. | Not listed. | Not listed. | Experimenters | No |
| Swenson-Pierce et al. 1987 [8] | To improve independent performance of domestic tasks. | To learn and effectively use instructional techniques to increase prompting and social praise in teaching a domestic task to their sibling with NDD. | Sessions for 2-3 times per week, for approximately 3 hours. The initial training session was completed within an hour. All sessions were at home. | The training procedure for preparing the siblings to act as instructors for their brother or sister with a disability consisted of the following components: (a) an explanation of their role as an instructional agent, (b) an overview and discussion of the system of increased prompting and social praise, (c) an overview and discussion of the task analysis, (d) a period of role playing where the instructor portrayed as the sibling with NDD, and (e) a period of in vivo instruction accompanied by instructor feedback. | Not listed. | Not listed. | Instructor. | Yes |
| Clark et al. 1989 [9] | To enhance social interaction between siblings with and without autism spectrum disorder. | To train siblings to enhance their social interaction with their sibling with autism spectrum disorder. | Nineteen 2-hour sessions at clinic. | During baseline, siblings were taught a general problem-solving strategy to introduce the children to role playing and problem-solving discussion format. The children were asked to generate alternative solutions and to evaluate their potential effectiveness. The therapists then role played these ideas. Siblings were then prompted to evaluate their suggestions and to practice these strategies in role playing with the therapist. | Not listed. | Not listed. | Therapist. | No |
| Craft et al. 1990 [10] | Not listed. | To promote spontaneous activity among siblings, which could help children with cerebral palsy. | Twice a month, for four months at home. | Two phases: educational and reinforcement of learning. Educational content was divided into discussions of cerebral palsy (e.g., “What is cerebral palsy?”) and promoting motivational skills (“What can I do to help my brother or sister be more independent?”). Learning reinforcement activities focused on ways to increase the functional skills of the siblings with cerebral palsy. Activities for recreational and for the siblings to share feelings and problems were included. | Not listed. | Not listed. | Study investigators | No |
| Coe et al. 1991 [11] | To target behaviours functional manipulation of play materials and activity related verbalizations (i.e., verbal requests for play materials, compliments to siblings, comments or descriptions of play activity). | To target behaviours, including appropriate use of verbal prompts, nonverbal prompts, verbal reinforcement and tangible reinforcement to shape behaviour. | Treatment sessions began with a five-minute training period. Sessions were conducted for 2 to 3 afternoons a week for approximately 15-20 weeks. All sessions were conducted at home. | Training of: 1) nonverbal play behaviour (assembling Tinker Toy pieces or rolling a truck, depending on the child-sibling pair) and 2) verbal behaviour (requesting the Tinker toy piece or the truck). Nonverbal prompts taught to the sibling include 1) use of manual guidance of the head to establish attention; 2) guidance of hands in executing nonverbal play responses; 3) response interruption of stereotypes and inappropriate responses such as throwing toys or stealing reinforcers. Verbal prompts taught included: 10 activity orientation ("We're going to play with the tinker toys (truck) today and earn stickers and chips"); 2) direction and behaviour ("Say 'Stick please!'"; "Say 'I want the truck, please' for a chip;". Reinforcement training encompassed delivery of both verbal praise and tangible reinforcers (e.g., sticker or edible). | Not listed. | Not listed. | Trainer and undergraduate students. | No |
| Celiberti et al.1993 [12] | Not applicable. | To provide training of a generalized set of behavioural skills to siblings. | 15-minute training session for 13-17 sessions at home. | The training curriculum included: how to deliver play-related commands effectively, how to use social praise for appropriate responses, and how to respond when the child with autism did not comply with a request. The trainer demonstrated the behavioral skills for a maximum of 5 minutes with the sibling with autism spectrum disorder while the sibling observed. The trainer discussed the various skills and procedures to be acquired. For the next 10 minutes the sibling practiced the behavior with the child with autism, while the trainer offered feedback. If necessary, the sibling role-played with the trainer behaviors that were causing difficulty. The sibling was trained successively in each of the three sets of skills until she reached the criterion of 80% correct responding over two consecutive sessions for each set. | Not listed. | Not listed. | The first author, a graduate student in clinical psy-chology with over two years of intensive experience with children with autism spectrum disorder. | Yes |
| Hancock et al. 1996 [13] | To improve the siblings' language use at home during play and snack activities. Child language behaviors consisted of (a) frequency of total child utterances, (b) frequency of targets used spontaneously, and (c) frequency of total use of targets (prompted plus spontaneous). Table 2 provides definitions. | To train siblings to teach two milieu teaching procedures, modeling and mand modeling. | 45-minute training sessions, for a total of four sessions at home. | Training consisted of: presenting the sibling with a written manual that had been designed specifically to fit each sibling's interests and reading comprehension level (assessed informally based on grade level), discussing the material presented in the manual with the sibling, watching videotapes modeling the milieu teaching techniques with children who exhibited language delays, watching videotapes of the sibling and sibling with NDD recorded during baseline and having the sibling identify appropriate instances for applying the milieu technique, role playing use of the milieu techniques with the adult trainer, and engaging in practice sessions with the sibling and sibling with NDD with feedback. | Not listed. | Modification of training package previously used with parents. | First author, a female with masters-level preparation in child development and 3 years experience implementing milieu teaching procedures with young children. | No |
| Trent et al. 2005 [14] | To improve the communication behaviour. | To teach two responsive interaction strategies (mirroring and verbal responding). | 30–60-minute sessions, two sessions per week, with each, approximately 35 sessions in total at home. | The investigator taught the sibling one of the responsive interaction strategies or reviewed the previously taught strategies. The teaching and reviewing portion of the intervention consisted of five components: presentation of information by the investigator with a manual, opportunity for the sibling to discuss the procedures and ask questions, use of modeling and role play to practice using the strategies, a second opportunity to discuss and ask questions, and setting up the activity for the play session. | Not listed. | Not listed. | Investigator and trainer | No |
| Tsao et al. 2006 [15] | To increase social participation. | To learn and use social skills strategies for interacting with their siblings with autism spectrum disorder. | About 10 sessions, for 10 minutes each at home. | Lessons taught common behavioral strategies for facilitating social interactions with the siblings with autism spectrum disorder, such as establishing eye contact, suggesting play activities, initiating conversations, or offering or asking for help. The training involved the researcher reviewing the previous day’s lesson, introducing the new skill, and reading a story to illustrate the use of the skill. The researcher modeled examples of new behaviours and parents could help to explain the story and behaviour. | Not listed. | Based on literature. | Researcher (the first author) | Yes |
| Stewart et al. 2007 [16]  Behavioural skills training | To improve conversation skills, with special emphasis on proper eye contact, soliciting input from the conversational partner regarding his or her interest in the topic, and avoiding topics on which the sibling with autism spectrum disorder typically perseverated. | To learn how to implement behaviour skills training to teach social skills. | 60 sessions. | Behaviour skills training was provided, comprised of instructions, modeling, rehearsal, and feedback. Instructions included a rationale for treatment, a description of the treatment process, and a clear presentation of the target behaviour. The target behaviour was modeled several times during various practice scenarios, followed by opportunities to perform the target behaviour during various rehearsal scenarios with feedback on the performance. | Not listed. | Literature about behavioral skills training and principles of behaviour modification. | Graduate student therapist (first author) | No |
| Trent-Stainbrook et al. 2007 [17] | To improve the intentional communicative behavior. | To teach two responsive interaction strategies (mirroring and verbal responding). | 30–60-minute sessions, two sessions per week, 19 sessions in total at home. | Intervention sessions were divided into three segments during both conditions of intervention. The first segment included either the trainer teaching the older sibling one of the responsive interaction strategies or reviewing the previously taught strategies. There were four subcomponents to the teaching and reviewing portion of the intervention: (a) presentation of information by the trainer with the use of a Responsive Interaction Pictorial Manual developed for this project and available from the first author, (b) opportunity for the sibling to discuss the procedures and ask questions, (c) use of modeling and role play to practice using the strategies, and (d) a second opportunity to discuss and ask questions. | Not listed. | Adapted from previous studies. | First author and research assistant. | No |
| Tsao et al. 2010 [18]  Three-Step Sibling-Mediated Social Skills Intervention | To increase interactions between siblings and their sibling with a disability. | To learn strategies to elicit social interactions from their sibling with a disability, and learn ways to help their sibling with a disability become involved in play or social interactions. | Frequency and duration of intervention was not listed. The intervention was conducted at home and in the community. | The first one or two basic lessons help siblings learn how to use strategies consistently to elicit social interactions from their sibling with a disability. The subsequent lessons provide siblings some specific ways to help their brother or sister with a disability become involved in play or social interactions. | Not listed. | Based on a model. | Not listed. | No |
| Ferraioli et al. 2011 [19]  Joint Attention Intervention | To learn joint attention skills, which is defined as the ability to use "gestures and eye contact to coordinate attention with another person in order to share the experience of an interesting object or event". | To train siblings to implement a joint attention intervention with their sibling with autism spectrum disorder. | 15-minute training sessions two or three times per day, 1-2 times per week. Sessions ranged from 7-9 weeks at home. | Procedures of the intervention were reviewed with the sibling. The sibling then participated in a brief interactive instruction with the experimenter, including modeling and role-plays with feedback provided. Prompts were provided to the sibling when needed. | Not listed. | Based on previously described procedures from the literature. | Not listed. | Yes |
| Chu et al. 2012 [20] | To increase aquatic and social skills of both children with autism spectrum disorder and children. | To provide training to peers and siblings of individuals with autism spectrum disorder. | 60-minute sessions for 32 sessions in total over 16 weeks at a local indoor hydro-therapy and swimming pool. | A workshop was held to introduce the aquatic program, and siblings were completed a training course. Rules and roles were discussed. Assisting steps were introduced: (a) physical interactions and (b) social interactions. The program consisted of warm-up activities, teacher instructions, group games, and cool down activities in each group, including the sibling-assisted group. | Not listed. | Based on literature. | Instructors had under-graduate degrees in physical education, 1-2 years of experience working with individuals with autism spectrum disorder, and an additional training course by the primary researcher. | No |
| Oppenheim-Leaf et al. 2012 [21] | To increase play interactions between siblings. | To teach how to get their sibling with NDD to play with them, how to get their sibling with NDD to share toys with them, how to provide play-related instructions, and how to find out what the sibling with NDD wanted to play. | 20-30 minutes of teaching periods for 40 sessions at home. | Sessions during the teaching phase always began with role-play and generalization probes for the current skill. Once all probes of the day were completed, the sibling was taught the current target skill through the use of the teaching interaction procedure. The teaching interaction procedure involved didactic teaching, modeling, and role-plays. | Not listed. | Not listed. | Teacher. | No |
| Walton et al. 2012 [22]  Reciprocal Intervention Training | To increase reciprocal imitation skills in a naturalistic social context. | To train children to implement reciprocal imitation training with their siblings with autism spectrum disorder. | 15-to-30-minute session per week, for 10 weeks at home. | The trainer used a manual written in child-friendly language and augmented with pictures depicting the intervention techniques. The trainer explained the technique and read through the relevant portion of the manual with the sibling, role-played the technique with the sibling while giving instruction and feedback, and demonstrated the technique with the sibling with autism spectrum disorder while explaining the actions. After each technique was taught, a poster depicting the technique was placed in the playroom to remind the sibling to use the technique. Feedback was provided to the sibling at certain periods. | Not listed. | Based on literature. | The trainer (first author) was a graduate-level student with about 2 years of experience working with young children with autism spectrum disorder and trained in the intervention. | Yes |
| Lewandowski et al. 2014 [23]  Comic Strip Conversation Intervention | To address sibling conflict and support Theory of Mind development through learning about the social and emotional factors that contribute to both conflict and resolution. | Same objective as the sibling with NDD. | 12 times for each phase, two phases in total at home. | The researcher introduced and explained the symbols dictionary in the first few sessions to remind the sibling and sibling with NDD of the talking and thinking bubbles that would be used in the session. After the participants showed understanding of the established conventions, the symbols dictionary was left on the table but was not reviewed unless the participants had questions. The activity was introduced by explaining that the researcher would help the sibling and sibling with NDD to write and draw while discussing a challenging situation. Both the sibling and sibling with NDD explained the events that took place during the challenging situation, and participated in the writing and drawing as much as possible. | Not listed. | Based on comic strip conversation intervention described in the literature. | Researcher (first author). | No |
| Özen 2015 [24] | Target skills were taught such as how to perform a behaviour independently, taking turns, or saying appropriate phrases. | To teach social interaction skills, including using effective prompts, taking turns, or reinforcing appropriate play behaviours with the sibling with autism spectrum disorder. | 29 sessions at home. | Activities including watching sample video clips to teach social interaction skills, having the researcher describe the social interaction behaviour, providing opportunities for the siblings to ask questions, and having the sibling and the sibling with autism spectrum disorder play together on the iPad. The researcher provided feedback on the behaviours from the sibling and sibling with autism spectrum disorder. | Not listed. | Not listed. | Researcher with a background of special education, and has 23 years of work experience with children with autism spectrum disorder and their families. | Yes |
| Kryzak et al. 2017 [25]  Behavioral Skills Training | To improve reciprocal interactions between siblings. | To improve self-management (goal setting, monitoring, and recruiting reinforcement) of the Stay-Play-Talk curriculum. | Weekly sessions for 14 weeks at home. | The researcher reviewed the self-management task analysis with the sibling along with that day's lesson. After introducing the lesson, the researcher modeled the responses described in the curriculum. The sibling then rehearsed the lesson with feedback from the researcher. Then, the sibling proceeded to play games with their sibling with autism spectrum disorder. | Not listed. | Modified curriculum described in the literature. | First author with a background in psychology and forensic sciences, and experience with applied behaviour analysis. | No |
| Neff et al. 2017 [26] | To learn on-task behavior. | To train siblings to provide positive reinforcement and prompts during play sessions with their sibling with autism spectrum disorder. | 3-5 min videos that are watched prior to 15-minute sessions at home and treatment center. | Customized videos were made for each pair of sibling and sibling with autism spectrum disorder. Each video consisted of at least three examples of physical prompting, gestural prompting, modeling prompting, and verbal prompting demonstrated by the researcher in the video. There were at least six examples of positive reinforcement modeled by the researcher across prompted and independent responses. The sibling watched the video corresponding to each game or activity immediately before playing the game with their sibling with autism spectrum disorder. Additional teaching was comprised of practice sessions with the researcher and feedback. | Not listed. | Not listed. | Researcher | No |
| Akers et al. 2018 [27] | To increase the number of appropriate vocalizations emitted by siblings with autism spectrum disorder. | To implement the script-fading procedure with their sibling with autism spectrum disorder. | Three sessions per day at home. Training ended when the sibling correctly implemented each component with their parent with 95% or better accuracy. The siblings met mastery after one session that took approximately 30 minutes. | The siblings were trained to: (a) orient to the sibling with autism spectrum disorder during play, (b) refrain from asking questions or giving directions, (c) respond to all of the verbalizations from their sibling with autism spectrum disorder, and (d) to comment on their own play actions. Siblings were instructed to present an auditory script and provide verbal prompts for their sibling with autism spectrum disorder to respond to. | Not listed. | Not listed. | Researcher. Parents of the siblings served as a research assistant. | No |
| Douglas et al. 2018 [28] | To increase sibling talk. | To increase sibling talk, as well as the frequency/percentage of responses to the sibling with complex communication needs. | One 45-min session and one 30-min session for a total of two training sessions, scheduled two days apart, conducted at home. | The first session provided a description of the strategy steps, visual text on a PowerPoint, video demonstration, and a handout of the training content. Verbal practice, questions, and application activities were included for each strategy step. The second session included a quiz of the strategy steps, role-play of the strategy during a play activity with the trainer, and a practice play session with feedback with their sibling with complex communication needs. | Not listed. | Based on the literature and adaptation of other sibling programs. | First author. | Yes |
| Spector et al. 2018 [29] | To increase imitative utterances, vocalizations, and speech. | To implement Natural Learning Paradigm using a video. | 20–25-minute training session; 5 minute sessions of sibling-mediated intervention for a total of 20 sessions, at an after school behavioural management center. | Siblings were trained to implement Natural Learning Paradigm using a video that role-modeled the strategies. Each sibling was told they will be taught to play a game with their sibling with autism spectrum disorder. The sibling was tested for comprehension by role-playing the procedures in the video. Siblings were then asked to play a game with their sibling with autism spectrum disorder using the strategies from the video. | Not listed. | Based on the literature and adaptation of other sibling programs. | Adult therapist. | No |
| Daffner et al. 2020 [30] | To increase the frequency of positive social behaviours and decrease the frequency of negative social behaviours. | To teach specific behaviour strategies and prompting skills for facilitating social interactions with the child with attention deficit hyperactivity disorder. | 15-30 minutes sessions twice a week for approximately 17-22 sessions in total at home. | Siblings were taught three lessons focused on specific social behavior strategies to help their brother or sister with attention deficit hyperactivity disorder in play or social interactions, such as sharing, giving or asking for help, and compromising. The researcher explained the strategy, used videos to demonstrate the strategy, and allowed the sibling to practice the strategy with feedback from the researcher. The sibling then played with their sibling with autism spectrum disorder after the training with no additional feedback. | Not listed. | Modification of peer-mediated intervention programs from the literature. | First author | Yes |
| Tsao 2020 [31] | To increase their social interactions with their siblings. | To learn and implement social skills strategies while playing with their siblings with developmental disabilities. | 15-20 minutes for 5-7 sessions in total at home. | The first two lessons introduced the three steps of the curriculum, and the following lessons focused on specific strategies to involve the siblings with developmental disabilities such as offering to help and requesting assistance. The researcher reviewed the previous lesson, introduced the skill/strategy, and read a story illustrating the use of the skill/strategy. After the training sessions, the researcher encouraged the siblings to use the discussed strategies when playing with their siblings with developmental disabilities. | Not listed. | Based on literature. | Researcher. | No |

Abbreviation: NDD, neurodevelopmental disabilities.

**References**

1. Weinrott MR. A training program in behavior modification for siblings of the retarded. Am J Orthopsychiatry. 1974;44:362–75.
2. Doleys DM, Slapion MJ. The reduction of verbal repetitions by response cost controlled by a sibling. J Behav Ther Exp Psychiatry 1975;6:61–3.
3. Miller NB, Cantwell DP. Siblings as therapists: a behavioral approach. Am J Psychiatry. 1976;133:447–50.
4. Colletti G, Harris SL. Behavior modification in the home: Siblings as behavior modifiers, parents as observers. J Abnorm Child Psychol. 1977;5:21–30.
5. Schreibman L, O’Neill RE, Koegel RL. Behavioral training for siblings of autistic children. J Appl Behav Anal. 1983;16:129–38.
6. Lobato D, Tlaker A. Sibling intervention with a retarded child. Educ Treat Children. 1985;8:221–8.
7. James SD, Egel AL. A direct prompting strategy for increasing reciprocal interactions between handicapped and nonhandicapped siblings. J Appl Behav Anal. 1986;19:173–86.
8. Swenson-Pierce A, Kohl FL, Egel AL. Siblings as home trainers: A strategy for teaching domestic skills to children. Journal of the Association for Persons with Severe Handicaps. 1987;12:53–60.
9. Clark ML, Cunningham LJ, Cunningham CE. Improving the social behavior of siblings of autistic children using a group problem solving approach. Child Fam Behav Ther. 1989;11:19–33.
10. Craft MJ, Lakin JA, Oppliger RA, Clancy GM, Vanderlinden DW. Siblings as change agents for promoting the functional status of children with cerebral palsy. Dev Med Child Neurol. 1990;32:1049–57.
11. Coe DA, Matson JL, Craigie CJ, Gossen MA. Play skills of autistic children: Assessment and instruction. Child Fam Behav Ther. 1991;13:13–40.
12. Celiberti DA, Harris SL. Behavioral intervention for siblings of children with autism: A focus on skills to enhance play. Behav Ther. 1993;24:573–99.
13. Hancock TB, Kaiser AP. Siblings’ use of milieu teaching at home. Topics Early Child Spec Educ. 1996;16:168–90.
14. Trent JA, Kaiser AP, Wolery M. The use of responsive interaction strategies by siblings. Topics Early Child Spec Educ. 2005;25:107–18.
15. Tsao LL, Odom SL. Sibling-mediated social interaction intervention for young children with autism. Topics Early Child Spec Educ. 2006;26:106–23.
16. Stewart KK, Carr JE, LeBlanc LA. Evaluation of family-implemented behavioral skills training for teaching social skills to a child with Asperger’s disorder. Clin Case Stud. 2007;6:252–62.
17. Trent-Stainbrook A, Kaiser AP, Frey JR. Older siblings’ use of responsive interaction strategies and effects on their younger siblings with down syndrome. J Early Interv. 2007;29:273–86.
18. Tsao LL, McCabe H. Why won’t he play with me?: Facilitating sibling interactions. Young Exceptional Children. 2010;13:24–35.
19. Ferraioli SJ, Harris SL. Teaching joint attention to children with autism through a sibling-mediated behavioral intervention. Behavioral Interventions. 2011;26:261–81.
20. Chu CH, Pan CY. The effect of peer- and sibling-assisted aquatic program on interaction behaviors and aquatic skills of children with autism spectrum disorders and their peers/siblings. Res Autism Spectr Disord. 2012;6:1211–23.
21. Oppenheim-Leaf ML, Leaf JB, Dozier C, Sheldon JB, Sherman JA. Teaching typically developing children to promote social play with their siblings with autism. Res Autism Spectr Disord. 2012;6:777–91.
22. Walton KM, Ingersoll BR. Evaluation of a sibling-mediated imitation intervention for young children with autism. J Posit Behav Interv. 2012;14:241–53.
23. Lewandowski JF, Hutchins TL, Prelock PA, Murray-Close D. Examining the benefit of including a sibling in story-based intervention with a child with Asperger Syndrome. Contemporary Issues in Communication Science and Disorders. 2014;41:179–95.
24. Özen A. Effectiveness of siblings-delivered ipad game activities in teaching social interaction skills to children with autism spectrum disorders. Educ Sci: Theory Pract. 2015;15:1287–303.
25. Kryzak LA, Jones EA. Sibling self-management: Programming for generalization to improve interactions between typically developing siblings and children with autism spectrum disorders. Dev Neurorehabil. 2017;20:525–37.
26. Neff ER, Betz AM, Saini V, Henry E. Using video modeling to teach siblings of children with autism how to prompt and reinforce appropriate play. Behavioral Interventions. 2017;32:193–205.
27. Akers JS, Higbee TS, Pollard JS, Reinert KS. Sibling-implemented script fading to promote play-based statements of children with autism. Behav Anal Pract. 2018;11:395–9.
28. Douglas SN, Kammes R, Nordquist E, D’Agostino S. A pilot study to teach siblings to support children with complex communication needs. Commun Disord Q. 2018;39:346–55.
29. Spector V, Charlop MH. A sibling-mediated intervention for children with autism spectrum disorder: Using the Natural Language Paradigm (NLP). J Autism Dev Disord. = 2018;48:1508–22.
30. Daffner MS, DuPaul GJ, Kern L, Cole CL, Cleminshaw CL. Enhancing social skills of young children with ADHD: Effects of a sibling-mediated intervention. Behav Modif. 2020;44:698–726.
31. Tsao LL. Brothers as playmates for their siblings with developmental disabilities: A multiple-baseline design study. Child Youth Care Forum. 2020;49:409–30.
