# Supplementary material for: Programs to Prepare Siblings for Future Roles to Support Their Brother or Sister with a Neurodevelopmental Disability: a Scoping Review
Source: Curr Dev Disord Rep. 2023 Feb 21;10(1):47–79. doi: 10.1007/s40474-023-00272-w (PMC9942034; doi:10.1007/s40474-023-00272-w)
Supplement: Supplementary file 6 — Supplementary file6 (DOCX 126 KB) [file 40474_2023_272_MOESM6_ESM.docx]

**Supplementary File 6.** Outcomes of programs focused on empowerment, and key findings for the siblings.

| **Study** | **Program Outcomes** | **Key findings for the siblings without NDD** | **Key findings for the siblings with NDD** |
| --- | --- | --- | --- |
| Weinrott 1974 [1] | Knowledge acquisition, skill development, satisfaction with the program, empowerment. | Moderate or vast improvement in the quality of their interaction with their sibling with an intellectual disability. In nearly two-thirds of the families, siblings were observed to be spending more time with their sibling with an intellectual disability. All siblings, with one exception, placed demands upon their sibling with an intellectual disability to use newly acquired speech, to identify concepts, and to follow directions. Siblings exhibited more patience than before. Four siblings began serving as aides in their sibling with an intellectual disability in the classroom at school. Another two siblings selected special education as their prospective major in college, which was not made before the program and for one of the sibling, it was not considered. | Not listed. |
| Doleys et al. 1975 [2] | Implementation of a program by the sibling without NDD | This program could be designed and successfully carried out by the sibling in a natural environment. | The verbal behaviour of an adolescent with intellectual disability could be modified with the technique of response cost contingency. |
| Miller et al. 1976 [3] | Skill development | Decreased family arguing and increased positive interactions between siblings. | Able to play alone for as long as 45 minutes., was more outgoing and assertive in family interactions. Positive effects were maintained 6-months after. |
| Colletti et al. 1977 [4] | Skill development | Siblings can modify the behaviour of their siblings with NDD. In Experiment 1, a sibling delivered contingent reinforcement which dramatically increased the ability to carry out the task (i.e., bead stringing by the sister with autism spectrum disorder). In Experiment 2, two siblings were able to work effectively with their brother with NDD on separate tasks. | Not listed. |
| Schreibman et al. 1983 [5] | Skill development | The siblings became proficient in behavioral teaching skills. | There were improvements in the correct responding. Skills could be applied in different environments, and in a much less structured type of interaction than during the training sessions. |
| Lobato et al. 1985 [6] | Skill development | The sibling was effective in teaching the sibling with NDD basic self-care skills. | The results of the second intervention on bedmaking were indicating a positive trend but were prematurely terminated due to a separate injury. No issues with noncompliance during structured training sessions with the sibling. |
| James et al. 1986 [7] | Skill development | The siblings easily acquired the requisite skills with increased initiations for interactions. | The baseline data for the handicapped siblings showed that their major deficit was initiations rather than responsiveness. Although the siblings with disabilities rarely initiated interactions with their siblings, they did respond appropriately to initiations made by their siblings.  The interactions between siblings generalized to larger play groups and across siblings. |
| Swenson-Pierce et al. 1987 [8] | Skill development | Successful training to use increased prompting and social praise with a high degree of accuracy. Enjoyment in participating in the study, which did not interfere significantly with personal time. Two siblings indicated that they used their skills when assisting their sibling with NDD with other tasks. | The siblings were able to perform the skills more independently. |
| Clark et al. 1989 [9] | Skill development, sibling attitude | Increased use of attending strategies and sign languages, and decreased use of controlling strategies. There was variation across sibling pairs with respect to the level of maintenance of these behaviors at follow-up. | Reductions in behavioral problem severity. Parents reported increases in positive interactions between siblings, which was maintained at six-months. |
| Craft et al. 1990 [10] | Knowledge acquisition and skill development | Increased self-confidence. | Increased family interactional patterns through self-reports and family functioning from parent reports. Indirect benefits of improved physical functioning. Reports that the siblings understood them better, spent more time with them, and were more patient with them. Some parents felt that siblings gained an increased sense of importance and came to know how much the sibling interactions meant to their family. |
| Coe et al. 1991 [11] | Skill development | Siblings quickly mastered both prompt and reinforcement techniques and were able to employ them without direct adult supervision. | Low frequency of verbal play behaviour and variable nonverbal play. By the end of the study, nonverbal and verbal play responses occurred in over 72% of recording intervals. |
| Celiberti et al. 1993 [12] | Skill development | Demonstrated rapidly mastery of target skills and sustained maintenance of skills as training shifted to other targeted areas and during follow-up probes, and able to apply skills to other types of interactions with the sibling with NDD (e.g., at dinnertime, outdoor activities). Became more comfortable interacting with sibling with autism spectrum disorder. | Not applicable. |
| Hancock et al. 1996 [13] | Skill development | All siblings learned the milieu language teaching techniques and implemented these procedures in a play setting with their siblings with NDD. They all reported that they enjoyed spending time with their siblings with NDD in a play situation and that they learned about their siblings’ competencies from the experience of being involved in the intervention. | When the teaching behaviors by the sibling, teaching continues to affect the language behaviors demonstrated by the siblings with NDD. |
| Trent et al. 2005 [14] | Skill development | Siblings learned the responsive interaction techniques quickly and used them in interactions with their younger siblings with Down syndrome during intervention. Siblings increased their use of mirroring and verbal responding during play interactions. When training for verbal responding was introduced to the target siblings, the number of intervals during which they used mirroring decreased. Responsive interaction strategies were maintained at 1-month follow-up. | Modest effects on their verbal behaviors and maintained at 1-month follow-up. |
| Tsao et al. 2006 [15] | Skill development | Moderate support for the effectiveness of a social intervention that involved siblings as mediators of social interactions for the siblings with autism spectrum disorder. The program did encourage more social interactions between siblings and their siblings with autism spectrum disorder. For three of the four siblings, social initiations toward their siblings with autism spectrum disorder increased during the program intervention phase. | Modest, positive changes in the social interactions of three siblings with autism spectrum disorder. Modest evidence for maintenance of social behavior. Clear increases in joint attention for three siblings. Limited evidence of social behaviour responses in other settings. |
| Stewart et al. 2007 [16] | Skill development | The sibling was able to prompt appropriate conversational behavior in the natural environment. | Appropriate eye contact was made during 78.6% of intervals, asked whether the partner was bored during 53.6% of intervals, asked whether the student assistant wished to change the topic during 53.6% of intervals, and always avoided perseverative topics. |
| Trent-Stainbrook et al. 2007 [17] | Skill development | Responsive interaction techniques were quickly learned and used them in interactions with the siblings with NDD. Use of these strategies were maintained at 1-month follow-up for all siblings. The effects of the intervention in the play setting did not readily generalize to the snack setting. Sibling interactions during generalization sessions were positive, but the siblings took very asymmetrical roles. The younger siblings often needed assistance preparing their own snacks. The older siblings often assumed the role of a teacher or helper, instructing the younger siblings on how to prepare the snack rather than being responsive to their acts of intentional communication. | Slight increase in the number of comments made in each session. No change occurred in the number of requests made by the younger siblings with Down syndrome. The quality of interactions between siblings appeared to improve. |
| Tsao et al. 2010 [18] | Skill development | Not applicable. | Not applicable. |
| Ferraioli et al. 2011 [19] | Skill development | Siblings implemented the intervention with high fidelity for most components, although it was difficult for them to remember all components without prompts (e.g., providing differential praise). Siblings were generally able to understand and apply concepts of obtaining and maintaining attention, providing tangible reinforcement, and persisting. All the siblings found the treatment acceptable by indicating during interviews that teaching was fun and that they would continue using the skills after the conclusion of the study. | There was meaningful change in the responding skills, with maintenance of acquired skills for responding for all siblings with NDD and initiating from some siblings with NDD. Performance reflected individual variation and response. |
| Chu et al. 2012 [20] | Skill development | Enhanced aquatic skills. | Increased physical and social interactions. |
| Oppenheim-Leaf et al. 2012 [21] | Skill development | Ability to demonstrate all skills taught with a teacher and the sibling with autism spectrum disorder. Play interactions between the sibling and sibling with autism spectrum disorder increased during free-play situations. Generalization of training to the free-play situation varied between siblings. | Performance of the siblings with autism spectrum disorder during generalization probes suggested that the siblings were effective at encouraging their siblings with autism spectrum disorder to engage in targeted social behaviours. Following the program, the siblings with autism spectrum disorder frequently engaged in the targeted social behavior, either independently or following a prompt from the sibling. |
| Walton et al. 2012 [22] | Skill development | Varying success learning to use the three intervention strategies independently. Reported enjoyment of the intervention, although the skills were a little hard to learn. | Some siblings with autism spectrum disorder showed increases in certain behaviours. None of the siblings with autism spectrum disorder maintained their skill gains when interacting with a different child. However, many of the sibling's gains were maintained (or even continued to increase) at the 1-month follow-up. |
| Lewandowski et al. 2014 [23] | Knowledge acquisition | No effect of intervention. | No effect of intervention. During the co-occurring intervention for both siblings, the sibling with NDD showed improvements in early and basic Theory of Mind competencies, which is the ability to read and interpret thoughts and feelings of self and others. According to parent report, the sibling with NDD also demonstrated the ability to better able negotiate challenging situations with his sibling. |
| Özen 2015 [24] | Skill development | Demonstrated ability to perform social interaction skills with 85-91% accuracy, which were maintained 1-week and 2-weeks after the program. Skills were generalizable to different children with autism spectrum disorder. | Siblings with autism spectrum disorder demonstrated varying abilities to acquire skills, including following directions, taking turns, responding appropriately to learning opportunities. Targeted skills were maintained 1-week and 2-week after the program. |
| Kryzak et al. 2017 [25] | Skill development | Improved self-management (goal setting, monitoring, and recruiting reinforcement) of a social skills curriculum.  Improvements were largely maintained and generalized to different settings. | Some corresponding improvements in reciprocal interaction of both siblings. Siblings with autism spectrum disorder also showed improved reciprocal interactions after intervention that were largely maintained through the 14-week maintenance sessions. |
| Neff et al. 2017 [26] | Skill development | Video modeling alone served as an effective teaching device for teaching prompting and reinforcement skills during play for two of the three siblings. Learned skills were generalizable to novel games. | When the siblings increased their use of appropriate prompts and reinforcement, the task behavior of their sibling with autism spectrum disorder subsequently increased. When supplemental reinforcement was introduced for the sibling, the delivery of appropriate prompts and reinforcement from the sibling increased, the task behavior of the sibling with autism spectrum disorder also increased. |
| Akers et al. 2018 [27] | Knowledge acquisition and maintenance of script fading procedures for the sibling with autism spectrum disorder at follow-up. Skill development. | Demonstrated ability to implement procedures with fidelity. | The number of contextually appropriate statements increased. |
| Douglas et al. 2018 [28] | Skill development | Increased frequency of sibling communication supports. | Increased communication for the siblings with complex communication needs. |
| Spector et al. 2018 [29] | Skill development | Demonstrated ability to effectively learn the training techniques from the Natural Learning Paradigm in a short amount of time and engage in these techniques with their sibling with autism spectrum disorder. | Two of three children with autism spectrum disorder showed increases in speech and verbalization. Generalization of treatment gains was limited. |
| Daffner et al. 2020 [30] | Satisfaction with the program from the perspectives of the parents and sibling participants. Skill development for the siblings. | All three siblings either agreed or strongly agreed that the strategies they learned to use were fair, the strategies helped them and the sibling with attention deficit hyperactivity disorder be better playmates, and learning to use the strategies was easy. Increased use of several positive social behaviours including sharing, asking/giving help, and compromising. Siblings were observed to successfully learn and use these specific social skill strategies with relatively high integrity. | Promoted positive social behaviours and social skills. |
| Tsao 2020 [31] | Skill development | Moderate support for the effectiveness of a social interaction intervention that involved siblings as mediators of social interactions for children with developmental disabilities. Medium effect on the application of strategies by siblings. The intervention did encourage more social interactions between brothers and children with developmental disabilities. | Some effect on the social behaviors of children with DD. There was a small effect on social behaviors of children with developmental disabilities and their siblings. |

Abbreviation: NDD, neurodevelopmental disabilities.

**References**

1. Weinrott MR. A training program in behavior modification for siblings of the retarded. Am J Orthopsychiatry. 1974;44:362–75.
2. Doleys DM, Slapion MJ. The reduction of verbal repetitions by response cost controlled by a sibling. J Behav Ther Exp Psychiatry 1975;6:61–3.
3. Miller NB, Cantwell DP. Siblings as therapists: a behavioral approach. Am J Psychiatry. 1976;133:447–50.
4. Colletti G, Harris SL. Behavior modification in the home: Siblings as behavior modifiers, parents as observers. J Abnorm Child Psychol. 1977;5:21–30.
5. Schreibman L, O’Neill RE, Koegel RL. Behavioral training for siblings of autistic children. J Appl Behav Anal. 1983;16:129–38.
6. Lobato D, Tlaker A. Sibling intervention with a retarded child. Educ Treat Children. 1985;8:221–8.
7. James SD, Egel AL. A direct prompting strategy for increasing reciprocal interactions between handicapped and nonhandicapped siblings. J Appl Behav Anal. 1986;19:173–86.
8. Swenson-Pierce A, Kohl FL, Egel AL. Siblings as home trainers: A strategy for teaching domestic skills to children. Journal of the Association for Persons with Severe Handicaps. 1987;12:53–60.
9. Clark ML, Cunningham LJ, Cunningham CE. Improving the social behavior of siblings of autistic children using a group problem solving approach. Child Fam Behav Ther. 1989;11:19–33.
10. Craft MJ, Lakin JA, Oppliger RA, Clancy GM, Vanderlinden DW. Siblings as change agents for promoting the functional status of children with cerebral palsy. Dev Med Child Neurol. 1990;32:1049–57.
11. Coe DA, Matson JL, Craigie CJ, Gossen MA. Play skills of autistic children: Assessment and instruction. Child Fam Behav Ther. 1991;13:13–40.
12. Celiberti DA, Harris SL. Behavioral intervention for siblings of children with autism: A focus on skills to enhance play. Behav Ther. 1993;24:573–99.
13. Hancock TB, Kaiser AP. Siblings’ use of milieu teaching at home. Topics Early Child Spec Educ. 1996;16:168–90.
14. Trent JA, Kaiser AP, Wolery M. The use of responsive interaction strategies by siblings. Topics Early Child Spec Educ. 2005;25:107–18.
15. Tsao LL, Odom SL. Sibling-mediated social interaction intervention for young children with autism. Topics Early Child Spec Educ. 2006;26:106–23.
16. Stewart KK, Carr JE, LeBlanc LA. Evaluation of family-implemented behavioral skills training for teaching social skills to a child with Asperger’s disorder. Clin Case Stud. 2007;6:252–62.
17. Trent-Stainbrook A, Kaiser AP, Frey JR. Older siblings’ use of responsive interaction strategies and effects on their younger siblings with down syndrome. J Early Interv. 2007;29:273–86.
18. Tsao LL, McCabe H. Why won’t he play with me?: Facilitating sibling interactions. Young Exceptional Children. 2010;13:24–35.
19. Ferraioli SJ, Harris SL. Teaching joint attention to children with autism through a sibling-mediated behavioral intervention. Behavioral Interventions. 2011;26:261–81.
20. Chu CH, Pan CY. The effect of peer- and sibling-assisted aquatic program on interaction behaviors and aquatic skills of children with autism spectrum disorders and their peers/siblings. Res Autism Spectr Disord. 2012;6:1211–23.
21. Oppenheim-Leaf ML, Leaf JB, Dozier C, Sheldon JB, Sherman JA. Teaching typically developing children to promote social play with their siblings with autism. Res Autism Spectr Disord. 2012;6:777–91.
22. Walton KM, Ingersoll BR. Evaluation of a sibling-mediated imitation intervention for young children with autism. J Posit Behav Interv. 2012;14:241–53.
23. Lewandowski JF, Hutchins TL, Prelock PA, Murray-Close D. Examining the benefit of including a sibling in story-based intervention with a child with Asperger Syndrome. Contemporary Issues in Communication Science and Disorders. 2014;41:179–95.
24. Özen A. Effectiveness of siblings-delivered ipad game activities in teaching social interaction skills to children with autism spectrum disorders. Educ Sci: Theory Pract. 2015;15:1287–303.
25. Kryzak LA, Jones EA. Sibling self-management: Programming for generalization to improve interactions between typically developing siblings and children with autism spectrum disorders. Dev Neurorehabil. 2017;20:525–37.
26. Neff ER, Betz AM, Saini V, Henry E. Using video modeling to teach siblings of children with autism how to prompt and reinforce appropriate play. Behavioral Interventions. 2017;32:193–205.
27. Akers JS, Higbee TS, Pollard JS, Reinert KS. Sibling-implemented script fading to promote play-based statements of children with autism. Behav Anal Pract. 2018;11:395–9.
28. Douglas SN, Kammes R, Nordquist E, D’Agostino S. A pilot study to teach siblings to support children with complex communication needs. Commun Disord Q. 2018;39:346–55.
29. Spector V, Charlop MH. A sibling-mediated intervention for children with autism spectrum disorder: Using the Natural Language Paradigm (NLP). J Autism Dev Disord. = 2018;48:1508–22.
30. Daffner MS, DuPaul GJ, Kern L, Cole CL, Cleminshaw CL. Enhancing social skills of young children with ADHD: Effects of a sibling-mediated intervention. Behav Modif. 2020;44:698–726.
31. Tsao LL. Brothers as playmates for their siblings with developmental disabilities: A multiple-baseline design study. Child Youth Care Forum. 2020;49:409–30.
